# Proteomic analysis of porcine mesenchymal stem cells derived from bone marrow and umbilical cord: implication of the proteins involved in the higher migration capability of bone marrow mesenchymal stem cells

**DOI:** 10.1186/s13287-015-0061-x

**Published:** 2015-04-15

**Authors:** Lei Huang, Chenguang Niu, Belinda Willard, Weimin Zhao, Lan Liu, Wei He, Tianwen Wu, Shulin Yang, Shutang Feng, Yulian Mu, Lemin Zheng, Kui Li

**Affiliations:** State Key Laboratory of Animal Nutrition and Key Laboratory of Farm Animal Genetic Resources and Germplasm Innovation of Ministry of Agriculture, Institute of Animal Sciences, Chinese Academy of Agricultural Sciences, No. 2 Yuanmingyuan West Road, Haidian District 100193, Beijing, China; The Institute of Cardiovascular Sciences and Institute of Systems Biomedicine, School of Basic Medical Sciences, and Key Laboratory of Molecular Cardiovascular Sciences of Ministry of Education, Peking University Health Science Center, No. 38 Xueyuan Road, Haidian District 100191, Beijing, China; Cleveland Clinic Lerner Research Institute Mass Spectrometry Laboratory for Protein Sequencing, Euclid Avenue, Cleveland, OH 44195 USA

## Abstract

**Introduction:**

Mesenchymal stem cells (MSCs) have the ability to proliferate *in vivo* with a large variety of differentiation potentials and therefore are widely used as an ideal material for cell therapy. MSCs derived from pig and human sources are similar in many aspects, such as cell immunophenotype and functional characteristics. However, differences in proteomics and the molecular mechanisms of cell functions between porcine bone marrow MSCs (BM-MSCs) and umbilical cord MSCs (UC-MSCs) are largely unknown. To the best of our knowledge, MSCs collected from different tissue have specific phenotype and differentiation ability in response to microenvironment, known as a niche.

**Methods:**

Porcine BM-MSCs and UC-MSCs were evaluated with flow cytometric and adipogenic and osteogenic differentiation analyses. We used isobaric tagging for relative and absolute quantitation (iTRAQ), combined with liquid chromatography-tandem mass spectrometry, to identify differentially expressed proteins (DEPs) between these two types of MSCs. Kyoto Encyclopedia of Genes and Genomes pathway and phenotype analyses were used to understand the links between cell migration ability and DEPs.

**Results:**

Two separate iTRAQ experiments were conducted, identifying 95 DEPs (95% confidence interval). Five of these proteins were verified by Western blotting. These 95 DEPs were classified in terms of biological regulation, metabolic process, developmental process, immune system process, reproduction, death, growth, signaling, localization, response to stimulus, biological adhesion, and cellular component organization. Our study is the first to show results indicating that porcine BM-MSCs have a higher migration capability than UC-MSCs. Finally, one of the DEPs, Vimentin, was verified to have a positive role in MSC migration.

**Conclusions:**

These results represent the first attempt to use proteomics specifically targeted to porcine MSCs of different tissues. The identified components should help reveal a variety of tissue-specific functions in tissue-derived MSC populations and could serve as important tools for the regeneration of particular tissues in future stem cell-based tissue engineering studies using animal models.

**Electronic supplementary material:**

The online version of this article (doi:10.1186/s13287-015-0061-x) contains supplementary material, which is available to authorized users.

## Introduction

Mesenchymal stem cells (MSCs), which are a type of adult stem cell developed from the mesoderm, can be isolated from the brain, liver, lung, kidney, fat, bone marrow, peripheral blood, umbilical cord blood, umbilical cord, placenta, amniotic fluid, and other tissues [1]. MSCs possess the potential for self-renewal and pluripotency and play an important role in tissue repair and regeneration [2]. When cultured *in vitro*, MSCs exhibit strong proliferation and can be induced to differentiate into bone, cartilage, adipogenic, and myogenic tissue; therefore, MSCs are widely used for bone and muscle repair in pre-clinical evaluation and clinical studies [1]. In addition, extensive clinical studies on MSC-based therapies have been conducted on many human diseases, including chronic graft-versus-host disease, systemic lupus erythematosus, cirrhosis, diabetes, acute kidney injury, and a variety of neurodegenerative diseases [1]. Bone marrow MSCs (BM-MSCs) are an important source of the adult stem cells widely used in basic and clinical research [3]. However, these cells are limited by the inconvenience of sample collection and by a reduced proliferation and differentiation capacity, coupled with effects of the donor’s age [2]. Therefore, searching for the detailed mechanism underlying these phenotypes and eliminating these limitations are important. In contrast, some studies have shown that umbilical cord MSCs (UC-MSCs) are similar to BM-MSCs regarding cell surface markers, physiological characteristics, proliferation and differentiation characteristics, and protein expression spectra; therefore, similar to BM-MSCs, UC-MSCs may be used for allogeneic stem cell transplantation in cell regeneration therapy [4]. MSCs that reside in different microenvironments have many similarities; however, a detailed comparison between BM-MSCs and UC-MSCs is required to verify their potential for use in clinical therapy.

In recent years, miniature pigs have been widely used as an animal model in stem cell biology, which is important for the treatment of human diseases and for xenotransplantation. Because the organ size, physiological level, genetic characteristics, and other aspects of the pig model are similar to those of humans, the findings on porcine stem cells may provide a theoretical basis and practical guidance for clinical applications in human diseases [5]. Therefore, it may be possible to use pig-derived stem cells as an alternative resource to treat human diseases. However, some researchers have shown that different sources of MSCs exhibited differences in gene expression patterns under specific culture conditions [3,6]. In addition, these cells could produce specific organizational structures depending on their growth environment and on their response to different ectopic microenvironments, thereby differentiating into different cell types [7]. Therefore, it is important to understand the molecular basis of these features and signaling mechanisms of MSCs for the clinical application of cell therapy. Analyzing gene expression levels and taking advantage of chip or sequencing technology would help us to understand the molecular mechanism of cellular functions; however, transcript level information may not completely overlap with protein levels [8]. Therefore, proteomic research would aid in understanding the molecular mechanism of MSC differentiation [6]. Compared with transcriptome data, proteome analysis could be used to study many aspects of the proteome, including protein expression levels, protein stability, subcellular localization, post-translational modifications, and protein interactions [8]. However, there are some limitations to proteomic approaches based on two-dimensional electrophoresis and mass spectrometry (MS) techniques. For example, detecting hydrophobic proteins and phosphorylated proteins is difficult [9]. These limitations have driven the development of new technologies for protein identification and quantitation. iTRAQ (isobaric tagging for relative and absolute quantitation) is a gel-free technique used in quantitative proteomic analysis that can identify low-abundance proteins [9]. This type of proteomic analysis may help researchers to understand the proteomic differences of diverse sources of MSCs.

A proteomic analysis of MSCs was first reported in 2001 [6]. Since then, MS-based proteomic technology has increasingly become an effective tool for detecting differentially expressed proteins (DEPs) of MSCs derived from various sources or in the state of *in vitro* differentiation [10]. Researchers have used proteomic technology to identify DEPs of human MSCs in the process of osteogenic differentiation [11]. In another study, rabbit BM-MSCs were induced by 5-azacytidine (5-aza) to differentiate into myocardial cells, and the resulting proteomic changes were analyzed [12]. Welsh *et al*. identified these up- and down-regulated proteins during adipogenic differentiation of MSCs [13]. However, MSCs derived from various sources exhibit differences in their protein expression profiles [6]. A proteomic comparison between BM-MSCs and UC-MSCs has already been reported for humans but not for large animals, such as pigs. Neng Seng *et al*. conducted a comparative analysis of the proteome of BM-MSCs in different generations isolated from Chinese miniature pigs only [14], and their research did not include a comparison of MSCs derived from different tissues. In the study of human MSCs, Guo Li *et al*. compared the proteome of BM-MSCs and UC-MSCs and then studied different proteins associated with cell migration [2]. Given the limitation in collecting human specimens, their experiment used a two-to-two design, and the two-dimensional electrophoresis-based MS and tandem MS (MS/MS) analysis detected only six DEPs between these two types of MSCs. Therefore, it is important to use an animal model, such as the pigs described above, and sensitive proteomic detection to uncover the differences between MSCs. In this study, we compared the DEPs from match-paired BM-MSC and UC-MSC samples, which were collected from the same piglets and cultured under identical conditions, and found 95 DEPs involved in different aspects of cell biology, especially cell motility. Additionally, iTRAQ quantitative proteomic techniques were used to clarify DEPs between BM-MSCs and UC-MSCs obtained from Wuzhishan mini-pigs. Accuracy of the results was assessed by Western blot. The results from these experiments indicate that the protein expression profiles of these two types of MSCs such as Vimentin, LGALS3, and TMSB4X are largely distinguishable in terms of cell motility.

## Methods

### Animals

The Animal Care Committee of the Chinese Academy of Agricultural Sciences approved all animal procedures. The newborn pigs were purchased from the National Germplasm Resources Center of Laboratory Miniature Pig.

### Isolation and culture of porcine mesenchymal stem cells

The MSCs were isolated from porcine umbilical cord (UC-MSCs) and bone marrow (BM-MSCs) as described previously. Briefly, the umbilical cords were collected when the piglets were born, and intact femurs were harvested from the same four Wuzhishan inbred pigs (WZSP) (Institute of Animal Sciences, Chinese Academy of Agricultural Sciences, Beijing, China) by sterile operation at 42 days after birth. The umbilical cord tissue was diced into 2- to 3-mm^3^ pieces, and the MSCs were separated by using a substrate-attached explant method. The bone marrow stem cells were extracted and centrifuged at 200 *g* for 5 minutes. The isolated MSCs were cultured in Dulbecco’s modified Eagle’s medium/F12 (DMEM/F12) (12500; Gibco, part of Life Technologies, Carlsbad, CA, USA) medium with 20% (vol/vol) fetal bovine serum (10099; Gibco), 50 units/mL penicillin G, and 50 μg/mL streptomycin and incubated at 37°C under 5% (vol/vol) CO_2_ in 100% humidified air. The media were changed every other day. The MSCs were harvested by digestion with 0.05% (wt/vol) trypsin-EDTA (25300054; Gibco) when the rate of cell fusion reached 80%. Cells were replanted in 100-mm dishes at a density of 1 × 10^4^/cm^2^.

### The evaluation of mesenchymal stem cells by flow cytometric analysis

The cultured MSCs were digested with 0.05% (wt/vol) trypsin-EDTA (Gibco), followed by washing with cold autoMACS Rinsing Solution (2°C to 8°C; Miltenyi Biotec, Bergisch Gladbach, Germany) three times. The pellets were resuspended in 1% (wt/vol) bovine serum albumin (Sigma-Aldrich, St. Louis, MO, USA) for 30 minutes at 4°C to block non-specific binding. Then, the UC-MSCs were incubated with rat anti-mouse CD31-APC (PECAM-1) (Miltenyi Biotec), mouse anti-human CD34-PE (Miltenyi Biotec), mouse anti-human CD45-PE (Miltenyi Biotec), or mouse anti-human CD90-FITC (Thy-1) (Abcam, Cambridge, MA, USA) monoclonal antibodies at 4°C for 30 minutes, respectively. The BM-MSCs were incubated with mouse anti-human CD29-FITC (Miltenyi Biotec), mouse anti-human CD34-PE (Miltenyi Biotec), rat anti-mouse CD44-FITC (Miltenyi Biotec), mouse anti-human CD45-PE (Miltenyi Biotec), or mouse anti-human CD90-FITC (Thy-1) (Abcam) monoclonal antibodies at 4°C for 30 minutes, respectively. The flow cytometric acquisition and data analysis were performed by using a BD FACSCalibur flow cytometer and Cell Quest software (BD Biosciences, San Jose, CA, USA). As a negative control, cells were incubated only with the corresponding isotype antibody, including rat IgG2a-APC (used for CD31; Miltenyi Biotec), mouse IgG2a-PE (used for CD34 and CD45; Miltenyi Biotec), rat IgG2b-FITC (used for CD44; Miltenyi Biotec), and mouse IgG1-FITC (used for CD29 and CD90; Miltenyi Biotec). These specimens could be placed in 4% paraformaldehyde for short-term preservation. Three independent flow cytometric experiments were performed.

### Adipogenic and osteogenic differentiation of mesenchymal stem cells

To evaluate MSC abilities, adipogenic and osteogenic differentiation assays were performed on isolated cells. Osteogenesis differentiation medium (Gibco) or adipogenesis differentiation medium (Gibco) was added into a culture when the fusion rate reached approximately 80%. The cells were cultured at 37°C in 5% (vol/vol) CO_2_ in 100% humidified air. The media were changed every 3 days, and the cells were cultured for 2 to 3 weeks before collection. Then, Alizarin Red S staining was used to analyze osteogenic lineages, whereas Oil Red O was used to analyze lipid droplets. Adipogenic and osteogenic differentiation assays were conducted three times for all four donor cells.

### Trypsin digestion and iTRAQ labeling

All the reagents and buffers required for iTRAQ labeling and cleaning were purchased from Applied Biosystems (Foster City, CA, USA). The iTRAQ labeling assay was conducted in accordance with the instructions of the manufacturer. Briefly, after digestion in culture flasks, total MSCs were collected and washed with cold phosphate-buffered saline (PBS) three times (2°C to 8°C). Cell pellets were directly used for extracting proteins or frozen in −80°C. The protein extraction process was carried out on ice in cold lysis buffer containing complete protease inhibitor cocktail (Roche, Basel, Switzerland). Proteins were stored in −80°C. Repeated freeze-thaw cycles were avoided. The proteins were dissolved in 8 M urea supplemented with 10 mM DTT, pH 8.5 (Amesco, St. Louis, MO, USA), and protein concentrations were determined by using the Bradford assay. Proteins were dissolved, denatured, alkylated, and digested with trypsin (Sigma-Aldrich; 1:20, wt/wt, 37°C for 18 hours). To label peptides with the iTRAQ reagent, 1 unit of label (defined as the amount of reagent required to label 100 μg of protein) was thawed and reconstituted in 70 μL of ethanol. The digestion reactions from UC-MSCs and from BM-MSCs were separately labeled with the 114 and 117 iTRAQ reagents, respectively. To identify more proteins, a strong cation exchange column (Applied Biosystems) was used to separate the mixed peptides. The elution buffers used were elution buffer A, containing 5 mM K_2_HPO_4_ in 20% (vol/vol) acetonitrile at pH 3.0, and elution buffer B, containing 5 mM K_2_HPO_4_ in 20% (vol/vol) acetonitrile and 350 mM KCl at pH 3.0. The labeled peptides were reconstituted in phase A and injected at a flow rate of 0.7 mL/minute into a high-resolution strong cation exchange (SCX) column (4.6 × 250 mm 5 μm; Thermo BioBasic, USA). After loading, the SCX column and C 18 precolumn were flushed with a three-step gradient sodium chloride solution (0, 50, and 100 mM) for 66 minutes. Then, the elution of the cation exchange groups was performed with an Agilent 1100 series high-performance liquid chromatography (HPLC) system, which was equipped with an autosampler, a 2/6 valve, and diode array detector (220 nm) (Agilent, Waldbronn, Germany), and 35 fractions were collected. Before liquid chromatography-MS/MS (LC-MS/MS), each fraction was desalted by using an SP-10 precolumn.

### Analysis by triple quadrupole time-of-flight tandem mass spectrometer

The eluted fractions were delivered into a nano reversed phase column (5-μm Hypersil C18 column, 75 μm × 150 mm; Thermo Fisher Scientific, Waltham, MA, USA) mounted in a Prominence Nano HPLC system (Shimadzu, Nakagyo-ku, Kyoto, Japan) and were eluted with an acetonitrile gradient from 5% to 40% containing 0.1% formic acid for 75 minutes at 400 nL per minute. The eluates were directly entered into a triple quadrupole time-of-flight (TOF) 5600 System (AB Sciex, Concord, ON, Canada), which was fitted with a Nanospray III source (AB Sciex) and with a pulled quartz tip as the emitter (New Objectives, Woburn, MA, USA), in positive ion mode and in a data-dependent manner, with full MS scan from 350 to 1,800 m/z.

The data were acquired by using an ion spray voltage of 2.5 kV, a curtain gas of 30 pounds per square inch (PSI), a nebulizer gas of 6 PSI, and an interface heater temperature of 150°C. The MS was operated with a resolving power of 30,000 full width at half maximum (FWHM) for TOF-MS scans. For information-dependent acquisition, survey scans were acquired in 250 milliseconds, and as many as 20 product ion scans were collected when exceeding a threshold of 125 counts per second, with a +2 to +5 charge state. A rolling collision energy setting was applied to all precursor ions for collision-induced dissociation. Dynamic exclusion was set at half the peak width (approximately 8 seconds), and then the precursor was refreshed off the exclusion list.

### Database searching and protein quantitation

In this study, we used ProteinPilot software 4.0 (AB Sciex, Foster City, CA, USA), including the Paragon™ and Pro Group™ algorithms, to interpret raw data files produced by MS. The parameters for searching were as follows: iTRAQ four-plex peptide labeled, trypsin digestion with only 1 maximum missed cleavage, carbamidomethylation for cysteine residues, variable oxidation for methionine, quadrupole TOF electrospray ionization, and identification, focusing on biological modifications. The tolerances were specified as ± 0.05 Da for peptides and ± 0.05 Da for MS/MS fragments. The National Center for Biotechnology Information and Swiss-Prot protein databases were chosen for searching, and the false discovery rate was controlled at 1% by using the integrated tools in ProteinPilot software. For protein assembling, the Pro Group algorithm was used to find the smallest number of proteins that could explain all the fragmentation spectral evidence.

Protein quantification was also performed by using ProteinPilot software, which automatically calculated the relative abundance of iTRAQ-labeled peptides and their corresponding proteins. Corrections were made for the impurity of iTRAQ reagents on the basis of the data provided by the manufacturer. For other similar errors in analyses, iTRAQ ratios were normalized by autobias, which used all data to calculate the bias correction factor.

### Gene Ontology annotation and enrichment analysis

DEPs were annotated by using the *Sus scrofa* Gene Ontology (GO) annotations database (updated on Jan. 2, 2014). The GO enrichment analysis was based on GO databases and was conducted by using tools displayed on the GO website. All the databases and tools were downloaded or linked from the website [15].

### KEGG pathway analysis

Pathway analyses of DEPs were based on the Kyoto Encyclopedia of Genes and Genomes (KEGG). The KEGG application programming interface and related databases were used to study the protein pathways. All of the resources were acquired from the website [16].

### Western blotting

The cells were washed with ice-cold PBS and lysed in mammalian protein extraction reagent (Thermo Scientific, Waltham, MA, USA) containing a protease inhibitor mixture (Roche Applied Science, Indianapolis, IN, USA). Cell lysates were centrifuged at 12,000 *g* for 10 minutes at 4°C, and the supernatant was collected. The protein concentrations were measured by using the Bradford protein assay with a Bicinchoninic Acid Protein Assay Kit (Thermo Scientific). In total, 20 μg of total protein was separated by 12% (wt/vol) SDS-PAGE and transferred onto a polyvinylidene fluoride (PVDF) membrane (Millipore, Billerica, MA, USA). Then, the membrane was blocked with 2% (wt/vol) bovine serum albumin (BSA) (Sigma-Aldrich) for 1 hour. Next, the previously indicated primary antibodies were used to probe the membrane overnight at 4°C. After extensive washing with Tris-buffered saline with Tween 20 (TBS-T), the membrane was incubated with secondary antibodies for 1 hour at room temperature. Bands were visualized by using SuperSignal West Pico Chemiluminescent Substrate (Pierce, part of Life Technologies) and recorded on x-ray films (Fuji Medical, Tokyo, Japan). Finally, the visualized bands were quantified by using Quantity One software on a GS-800 densitometer (Bio-Rad Laboratories, Hercules, CA, USA). The antibodies used were as follows: CNN1/calponin rabbit anti-human monoclonal (EP798Y) antibody (LifeSpan Biosciences, Inc., Seattle, WA, USA), Vimentin (D21H3) XP rabbit anti-human mAb (Cell Signaling Technology, Danvers, MA, USA), CTSB/cathepsin B rabbit anti-human polyclonal antibody (LifeSpan Biosciences, Inc.), TAGLN/SM22 rabbit anti-human polyclonal (C-terminus) antibody (LifeSpan Biosciences, Inc.), galectin-3/LGALS3 rabbit anti-human polyclonal antibody (Cell Signaling Technology), rabbit anti-mouse Akt polyclonal antibody (Cell Signaling Technology), Phospho-Akt (Ser473) (D9E) rabbit anti-human monoclonal antibody (Cell Signaling Technology), p44/42 MAPK (Erk1/2) (137 F5) rabbit anti-rat monoclonal antibody (Cell Signaling Technology), Phospho-p44/42 MAPK (Erk1/2) (Thr202/Tyr204) (D13.14.4E) rabbit anti-human monoclonal antibody (Cell Signaling Technology), and β-actin (13E5) rabbit anti-human monoclonal antibody (Cell Signaling Technology).

### RNA extraction and quantitative real-time polymerase chain reaction

Total RNA was extracted by using a MicroElute Total RNA Kit (Omega Bio-Tek, Norcross, GA, USA). The comparative delta-delta threshold cycle (Ct) method was adopted to analyze gene products by using the SYBR Select Master Mix (Applied Biosystems) in a 7500 Real-Time Polymerase Chain Reaction System (Applied Biosystems). Glyceraldehyde 3-phosphate dehydrogenase (*GAPDH*) was used as an internal control gene to calculate the relative gene expression. The experiment was repeated three times, and the results were expressed as the mean ± standard deviation. The primer sequences are reported in Additional file 1: Table S3.

### Short interfering RNA transfection

Stealth short interfering RNA (siRNA) (Invitrogen, part of Life Technologies) against *vimentin* was transfected into the BM-MSCs. Non-targeting siRNA was used as a control. The experiment was conducted in accordance with the protocol of the manufacturer (Invitrogen). Briefly, the cells were harvested and subcultured in antibiotic-free medium for 12 hours. Then, the cells were transfected when the rate of cell fusion reached 50%. The siRNAs were incubated with Lipofectamine 2000 (Invitrogen) for approximately 20 to 30 minutes and diluted with Opi-MEM medium (Gibco). The transfection mixture was gently added to the culture medium. Six hours later, the culture medium was replaced with fresh medium.

### Lentivirus-mediated overexpression in cell culture

UC-MSCs were plated in 60-mm dishes at a density of 5 × 10^5^ cells per dish. After 24 hours of cultivation, the cells were infected with a sham control lentivirus or lentivirus encoding porcine *vimentin* (LV5-NC or LV5-*vimentin*, respectively; GenePharma, Shanghai, China) at a multiplicity of infection of 100 in DMEM-F12 containing 10% fetal bovine serum (FBS). Twelve hours later, these cells were processed for Western blotting and migration analysis.

### Mesenchymal stem cell migration analyzed by a scratch assay and by a transwell migration assay

MSC migration was assessed by using a scratch assay and a transwell migration assay. The detailed process was as follows: Cells were cultured in 60-mm dishes to confluence and incubated with 10 μg/mL mitomycin-C for 2 hours. The growth-arrested cells were transferred into 24-well plates at a density of 8 × 10^4^ per well. Six hours later, ‘scratches’ were made along the bottom of the dish by using a 200-μL pipette tip. Then, the cells were cultured for another 48 hours. During incubation, dishes were placed under a phase-contrast microscope to acquire images at selected time points (6, 12, 24, 36, and 48 hours).

The cells were placed in the upper chamber of the transwell assembly (6.5-mm diameter inserts, 8.0-μm pore size; Corning Costar, Corning, NY, USA) with 100 μL of FBS-free medium at a density of 5 × 10^4^ or 2 × 10^5^ cells/mL in 200 μL, and 800 μL of medium containing 10% FBS as a source of chemoattractants filled the lower compartments. Cell number in the upper chamber of the transwell was not a coincidence among the three series of migration assays. For example, the cell number was 4 × 10^4^ per well in the migration assay of wild-type BM-MSCs and UC-MSCs or Vimentin-overexpressed BM-MSCs, respectively. However, in the migration assay of Vimentin-knocked-down UC-MSCs, the cell number was only 1 × 10^4^. After incubation at 37°C for 12 hours, the membrane was stained with Hoechst 33342 (Beyotime Institute of Biotechnology, Haimen, China), and the number of migrating cells was determined by counting 10 random fields per well under a fluorescence microscope at 40× magnification. Experiments were performed in sets of three for each group.

## Results

### Isolation, culture, and cell marker detection of porcine mesenchymal stem cells

**Figure 1:**
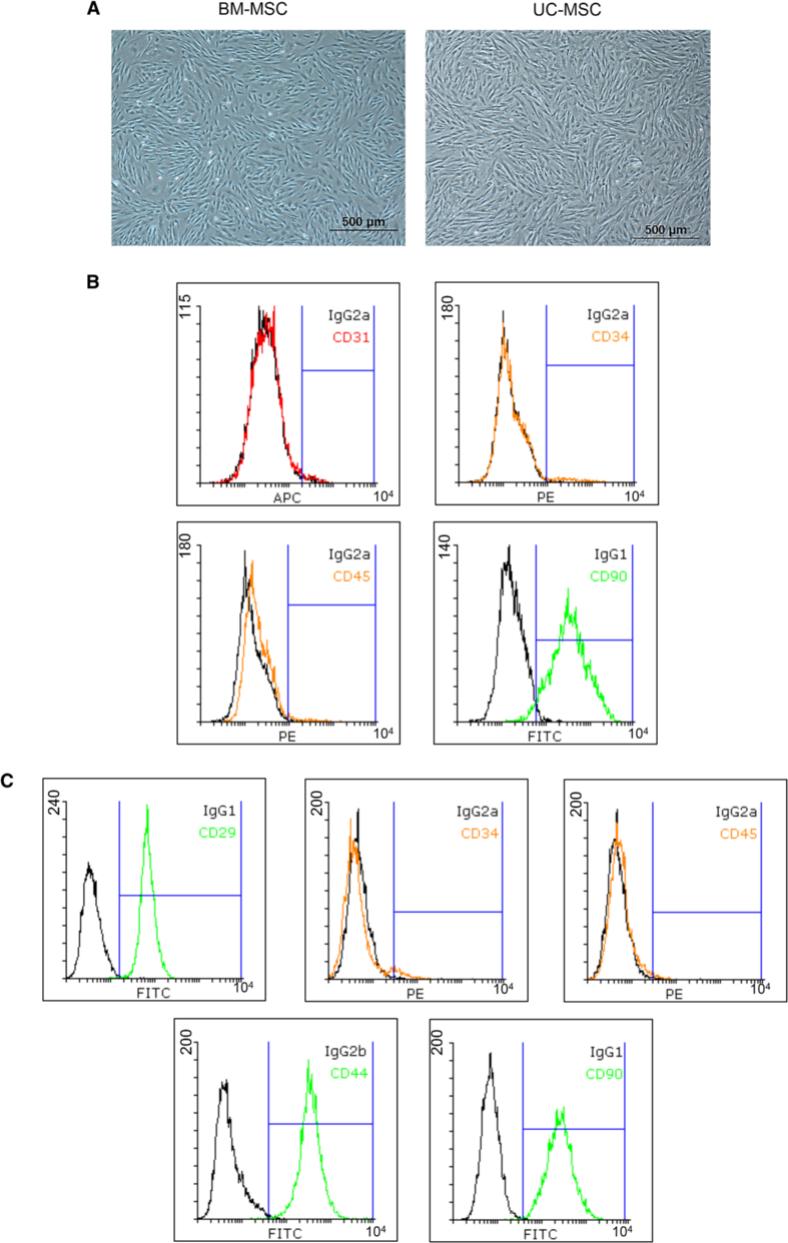
Mesenchymal stem cells were spindle-like and expressed certain protein markers on the cell surface. **(A)** Cell shape and growth states were similar between bone marrow mesenchymal stem cells (BM-MSCs) and umbilical cord mesenchymal stem cells (UC-MSCs) *in vitro*. **(B)** As shown in the overlay histograms, five cell surface markers of BM-MSCs were verified: CD29 (FITC, green), CD44 (FITC, green), and CD90 (FITC, green), which were positive, and CD34 (PE, orange) and CD45 (PE, orange), which were negative. **(C)** Four cell surface markers of UC-MSCs were verified: CD90 (FITC, green), which was positive, and CD31 (APC, red), CD34 (PE, orange), and CD45 (PE, orange), which were negative. FITC, fluorescein isothiocyanate; PE, phycoerythrin.

The cell type and growth state of porcine BM-MSCs were similar to those of UC-MSCs (Figure 1A). The supernatant of porcine UC-MSCs was plated in cell culture dishes. The cells were generally spindle-like and of different sizes, and the nucleus could not be identified. After 48 hours of cultivation *in vitro*, a few cells climbed from the edges of adherent tissue. During cultivation, the cells displayed colony growth, and most of the cells were spindle-like, although there were several polygonal-like cells as well. When the proliferating cell community integrated, the cells were arranged directionally, showing vortex-like growth (Figure 1A).

The purity of UC-MSCs and BM-MSCs was determined by flow cytometry. Cells with fluorescence in the range of M1 were considered positive cells, which were recognized by antibody detection. The auto-fluorescence intensity was less than 10^1^. The cells detected in this range were negative. Overlay histogram of each cell marker and its isotype antibody was made with Flowing Software (version 2.5.1). UC-MSC verification was performed by surface antigen expression, including the following four markers: positive for CD90, negative for vascular endothelial cell marker antigen CD31, negative for hematopoietic stem cell marker antigen CD34, and negative for leukocyte marker antigen CD45 (Figure 1B) (Additional file 1: Figure S3). The BM-MSC verification included the following five markers: positive for CD29, CD44, and CD90 and negative for CD34 and CD45 (Figure 1C) (Additional file 1: Figure S4).

### Porcine mesenchymal stem cells can differentiate into adipocytes and osteoblasts by *in vitro* induction

**Figure 2:**
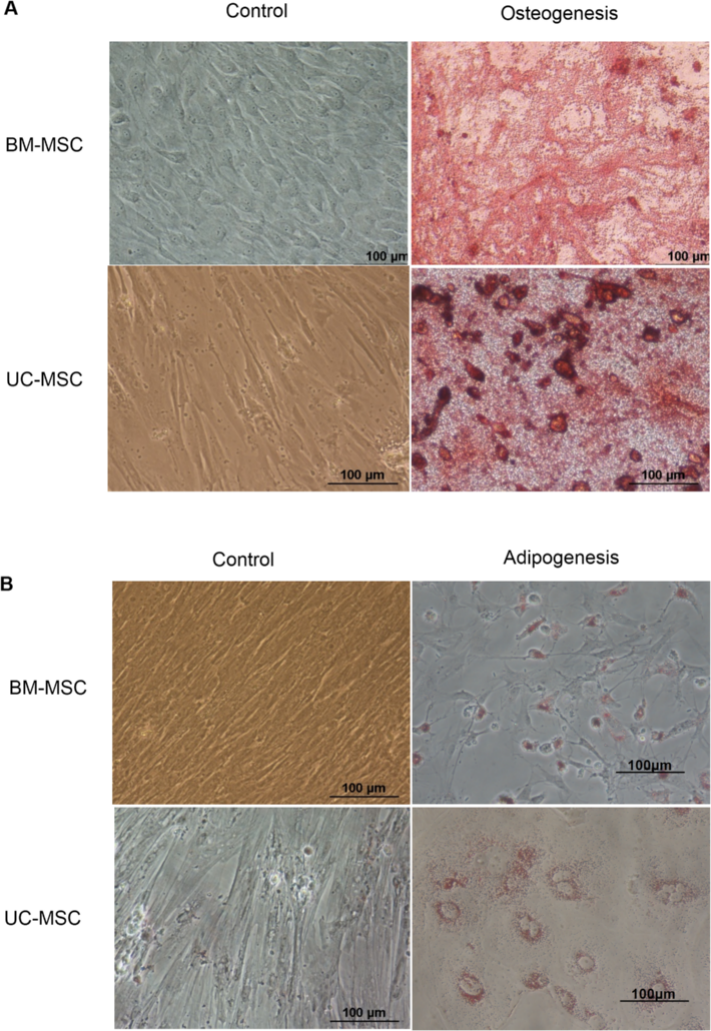
Mesenchymal stem cells could differentiate into adipocyte osteoblasts by induction *in vitro*. **(A)** Osteogenesis of bone marrow mesenchymal stem cells (BM-MSCs) and umbilical cord mesenchymal stem cells (UC-MSCs). After osteogenic induction, many calcium deposits appeared in osteocytes, which were stained red with Alizarin Red. **(B)** Adipogenesis of BM-MSCs and UC-MSCs. The lipid droplets generated in adipocytes were stained red with Oil Red O. Scale bars, 100 μm.

Osteogenic MSCs were stained with Alizarin Red. Clumps or a sheet of orange-red precipitate appeared in the intercellular space and were regarded as calcium nodules on the cell surface (Figure 2A). Adipogenic MSCs were stained by Oil Red. Orange fat particle drops in cells were regarded as fat synthesized during differentiation (Figure 2B). These results indicate that porcine MSCs have the ability to differentiate *in vitro*. The osteogenic and adipogenic differentiation potentials were used as the evaluation criteria of MSCs in our work. We did not conduct any quantitative assessment of osteogenic, adipogenic, and chondrogenic differentiation.

### Analysis of differentially expressed proteins detected in iTraqs and gene ontology enrichment analyses of these proteins

**Figure 3:**
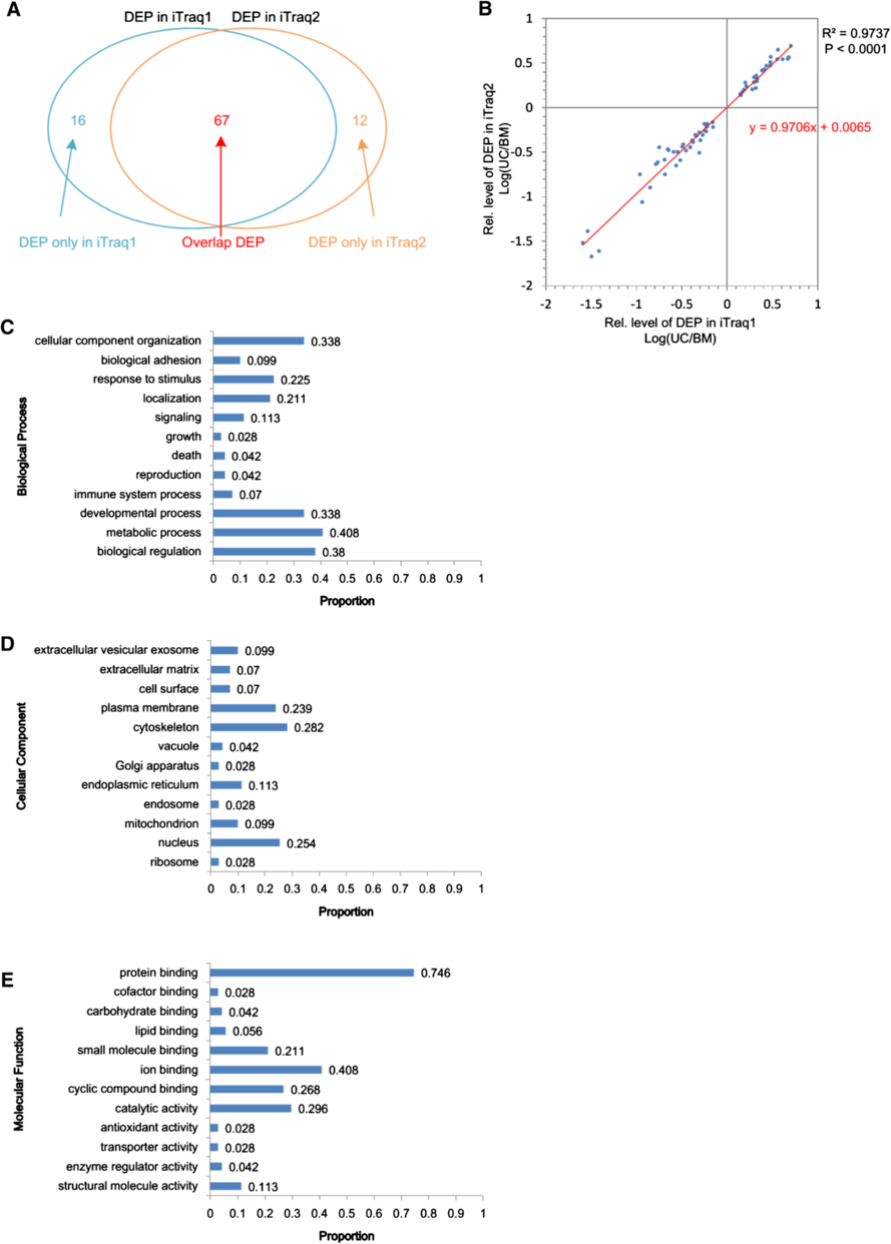
Summary of differentially expressed proteins (DEPs) detected in two iTraq experiments and Gene Ontology enrichment analyses of DEPs. **(A)** Overview of DEPs in two iTraq experiments. **(B)** An x-y scatter plot used to analyze the consistency of two sets of iTraq data, whereby the x-axis represents the log value of the protein level in the UC-MSC-to-BM-MSC (UC/BM) ratio in the first mass spectrometry detection, and the y-axis represents that parameter in the second detection. Histograms showing DEP enrichment in biological processes **(C)**, cellular components **(D)**, and molecular functions **(E)**. The enrichment degree was described by the ratio of the number of targeted proteins to all the annotated proteins. BM-MSC, bone marrow mesenchymal stem cell; iTraq, isobaric tagging for relative and absolute quantitation; UC-MSC, umbilical cord mesenchymal stem cell.

The samples were detected by using two independent iTRAQ-MS replicates. The DEPs were identified as proteins that had an absolute value of the log of the ratio of protein expression levels in UC-MSCs compared with BM-MSCs greater than 0.144 (specifically, the protein level between UC-MSCs and BM-MSCs differed by at least 39%). As a result, 83 and 79 DEPs were detected in the first and second MS replicates, respectively, and 95 DEPs were detected in total (Figure 3A and Table 1). There were five proteins whose expression levels between UC-MSCs and BM-MSCs were at least 10-fold different, namely, LGALS3, VIM, TMSB4X, CALD1, and P4HA1 (Table 1). In total, 67 DEPs were detected in both MS replicates, and the differential trend of these proteins is consistent between the replicates (Figure 3A and Table 1). In addition, the reproducibility of the differential protein levels between the technical replicates was assessed by using a scatter plot (Figure 3B). Briefly, the x-axis represents the log value of the iTRAQ ratios of the UC-MSCs to BM-MSCs in the first MS replicate, and the y-axis represents the iTRAQ ratios from the second replicate. This scatter plot can be fit linearly (Figure 3B) to obtain R^2^ and *P* values of 0.9737 and less than 0.0001, respectively. Of the 95 DEPs, 89 were annotated with GO terms by using the *Sus scrofa* GO annotations database and European Bioinformatics Institute Gene Ontology Annotation; we retrieved GO annotations of all of these proteins, and the annotations are listed in Additional file 1: Table S1. The GO enrichment analysis of biological processes, cellular components, and molecular functions showed that the enrichment degrees of DEPs in biological regulation, metabolic processes, developmental processes, immune system processes, reproduction, death, growth, signaling, localization, response to stimulus, biological adhesion, and cellular component organization are 0.38, 0.408, 0.338, 0.07, 0.042, 0.042, 0.028, 0.113, 0.211, 0.225, 0.099, and 0.338, respectively (Figure 3C). The degrees of DEP enrichment in the ribosome, nucleus, mitochondrion, endosome, endoplasmic reticulum, Golgi apparatus, vacuole, cytoskeleton, plasma membrane, cell surface, extracellular matrix, and extracellular vesicular exosome are 0.028, 0.254, 0.099, 0.028, 0.113, 0.028, 0.042, 0.282, 0.239, 0.07, 0.07, and 0.099, respectively (Figure 3D). The degrees of DEP enrichment in structural molecule activity, enzyme regulator activity, transporter activity, antioxidant activity, catalytic activity, cyclic compound binding, ion binding, small molecule binding, lipid binding, carbohydrate binding, cofactor binding, and protein binding are 0.113, 0.042, 0.028, 0.028, 0.296, 0.268, 0.408, 0.211, 0.056, 0.042, 0.028, and 0.746, respectively (Figure 3E).

**Table 1:** **Differentially expressed proteins detected by two iTraq experiments**

**UniProt accession number**	**Protein name**	**Gene name**	**Level in iTraq1 log(UC/BM)**	**Level in iTraq2 log(UC/BM)**
A3EX84	Galectin	*LGALS3*	−1.50000007	−1.66800008
P02543	Vimentin	*VIM*	−1.59599994	−1.51599999
B3XXC3	N/A	*TMSB4X*	−1.41600004	−1.60800006
F1SNH3	N/A	*CALD1*	−1.54000006	−1.38400002
A1X898	N/A	*P4HA1*	−0.93999986	−1.05999998
F1SU97	N/A	*PSAP*	−0.94400011	
F1SKI0	N/A	*MYH11*	−0.85200015	−0.89600002
F1SK03	N/A	*ANPEP*	−0.96399985	−0.7479999
Q56VQ1	N/A	*Oas2*	0.827999957	
F1SEQ7	N/A	*FAM213A*		0.772000048
F2Z5M2	N/A	*LOC100515138*	−0.68800004	−0.7479999
F1RWW4	N/A	*PDLIM5*	−0.78800002	−0.632
Q2YGT9	60S ribosomal protein L6	*RPL6*	0.70400004	0.695999967
D0G7F7	N/A	*TPM4*	−0.76800012	−0.61199992
F1RQW2	N/A	*C4*		−0.68800004
A9YUA9	N/A	*N/A*	−0.692	−0.58400011
A9GYW6	N/A	*APLE*	−0.63599995	
B2LUG8	N/A	*N/A*	0.680000019	0.568000024
F1RKW9	N/A	*MYO1D*	0.676000031	0.559999932
F1SDX6	N/A	*TGM2*	0.667999997	0.548000035
F1S663	N/A	*LAMC1*	0.559999932	0.652000004
F1SKJ1	N/A	*MYH9*	−0.56399995	−0.64799991
Q9TSX9	Peroxiredoxin-6	*PRDX6*	−0.752	−0.44400006
F1RGS2	N/A	*GBA*	0.612000045	0.544000055
F1SLI3	Microtubule-associated protein	*MAP4*	−0.64799991	−0.47600007
F1SMN1	N/A	*CALU*	−0.65599994	−0.46399992
F2Z557	N/A	*PABPC1*	−0.51999998	−0.58800002
C6K7I0	Importin subunit alpha	*N/A*	0.551999967	0.544000055
P20112	SPARC	*SPARC*	−0.59199998	−0.496
P27594	Interferon-induced GTP-binding protein Mx1	*MX1*	0.480000049	0.572000047
F1S4Y8	N/A	*LOC100621044*		−0.52399996
F1RSC3	N/A	*SCPEP1*		0.52399994
C0LZL0	Fascin	*FSCN1*	−0.548	−0.496
F1RJL6	N/A	*CLIP2*	−0.51199996	
F1SAP4	N/A	*WARS*		−0.5080001
Q08092	Calponin-1	*CNN1*		0.508000041
F1RST0	N/A	*HSPH1*	0.476000051	0.512000015
F2Z5K2	Proteasome subunit alpha type	*PSMA5*	0.476000051	0.476000051
F1RRV6	N/A	*NDRG1*	−0.45599997	−0.48000003
F1SJS8	N/A	*TAGLN*	−0.496	−0.43599995
F1SLA0	ATP synthase subunit beta	*ATP5B*	0.431999947	0.47199996
F1SQ11	N/A	*EEA1*	−0.48799998	−0.41200004
F1S764	N/A	*CPT2*		0.427999945
A5A768	N/A	*AP3D1*	0.427999945	
F1S554	N/A	*PALMD*	−0.40800001	−0.44400006
F1SGP8	N/A	*RCN1*	−0.40800001	−0.43999996
F1SMN5	N/A	*FLNC*	0.41199995	0.431999947
F1S827	N/A	*SERBP1*	−0.41599992	
F1SMV6	N/A	*LOC100737174*	−0.30800003	−0.50399997
A8CYB8	N/A	*RIG-I*	0.384000008	0.420000017
F1SIJ9	N/A	*PSAT1*	−0.39999997	
F1SV06	N/A	*PABPC4*	−0.37600003	−0.38400001
F1RKG8	N/A	*PEBP1*	−0.37600003	−0.38400001
F1S9A4	N/A	*NUCB2*	−0.38799997	−0.36800001
F2Z5C1	Annexin	*ANXA5*	0.360000074	
B6CVD6	N/A	*TXNDC4*	0.344000043	
F1SC51	N/A	*N/A*		0.344000043
F1RI39	N/A	*LOC100517284*	0.319999994	0.364000018
F1SPP8	N/A	*CKAP4*	−0.29599998	−0.36400003
F1SJJ5	N/A	*RPL4*	0.331999906	0.327999994
F1RJ93	N/A	*TAGLN2*	−0.33999999	−0.31599999
F1SS24	N/A	*FN1*	−0.35199996	−0.30400001
F1RRU7	N/A	*MRC2*	−0.34399997	−0.31199999
F1SGJ6	N/A	*SLMAP*	0.29600007	0.344000043
P33198	Isocitrate dehydrogenase [NADP], mitochondrial	*IDH2*	0.327999994	0.299999937
F1RWJ5	N/A	*KPNB1*	0.292000077	
F1S3M9	N/A	*EPB41L2*	0.29600007	0.288000032
F1SSA6	N/A	*LOC396903*	−0.30800003	−0.27600005
B0LY42	N/A	*N/A*	−0.27199999	−0.30400001
P28491	Calreticulin	*CALR*	−0.28400001	
Q4GWZ2	40S ribosomal protein SA	*RPSA*	0.315999943	0.224000024
B2CNZ7	N/A	*CTSB*		−0.26399998
F1SIX3	N/A	*UGP2*	−0.24799998	−0.25600002
F1SJR7	N/A	*TTLL12*	−0.23199998	−0.26399998
G9F6X8	N/A	*N/A*	0.275999909	0.208000125
F1RWT2	N/A	*PLS3*	0.199999944	0.284000063
B5L0Y2	N/A	*CAST*	−0.24000005	
F1RGP1	N/A	*MYBBP1A*	0.211999977	0.244000118
Q5MJE5	N/A	*N/A*		−0.22800001
Q06AT0	Hippocalcin-like protein 1	*HPCAL1*		−0.22800001
F1RI15	N/A	*HSPA4*	0.220000031	
F1SFZ8	N/A	*TLN1*	−0.22800001	−0.21199999
Q8SPT0	N/A	*N/A*		0.21600007
D0G0C6	Asparagine synthetase	*ASNS*	−0.224	−0.20799999
F1RS36	N/A	*HSPA5*	−0.25200001	−0.17999995
F1SQK1	N/A	*N/A*	−0.21999997	−0.17999995
F1S0V3	Annexin	*ANXA6*	0.183999971	0.203999991
F1SK12	N/A	*MAP1B*	−0.15599999	−0.21600001
F1SL58	N/A	*LOC100519091*	−0.18400006	
Q59IP2	N/A	*COL5A2*	−0.18400006	
Q29092	Endoplasmin	*HSP90B1*	0.167999844	0.179999932
F1S596	N/A	*PRKCSH*	−0.15999999	−0.15999999
P80021	ATP synthase subunit alpha, mitochondrial	*ATP5A1*	0.144000063	0.156000041
F1RR78	N/A	*LOC100049693*	0.151999844	0.144000063
B0LXK8	N/A	*HPRT1*	−0.144	

iTraq, isobaric tagging for relative and absolute quantitation; N/A, not applicable; UC/BM, umbilical cord mesenchymal stem cell/bone marrow mesenchymal stem cell.

### KEGG pathway analysis of differentially expressed protein indicated a remarkable difference in the motility capacity between UC-MSCs and BM-MSCs

**Figure 4:**
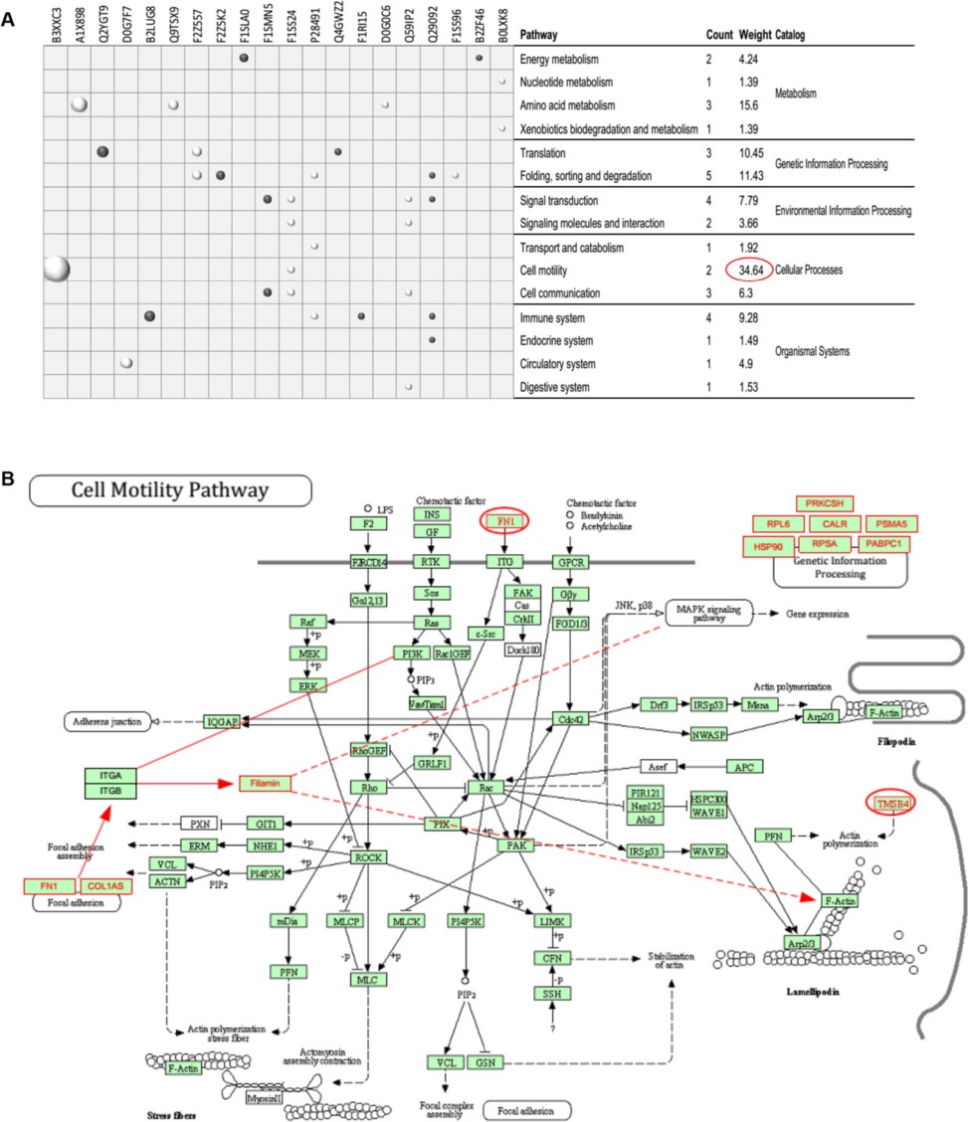
KEGG pathway analysis of DEPs indicated a remarkable difference in motility capacity between UC-MSCs and BM-MSCs. **(A)** A bubble graph describing the distribution of DEPs in a classified KEGG pathway. The bubble size represents the differential protein expression level. The dark bubbles show that the protein level in UC-MSCs is higher than that in BM-MSCs, whereas the light bubbles show the opposite trend. **(B)** A KEGG pathway map with additional manual annotations and links. The red boxes represent the DEPs detected in the experiment. The red boxes with red oval frames existed in the original KEGG pathway, and the red boxes with no oval frames and red links were added in line with a related KEGG pathway. BM-MSC, bone marrow mesenchymal stem cell; DEP, differentially expressed protein; KEGG, Kyoto Encyclopedia of Genes and Genomes; UC-MSC, umbilical cord mesenchymal stem cell.

In total, 20 of 95 DEPs were mapped by using the KEGG pathway database, and 37 pathways were obtained (Additional file 1: Table S2). The primary pathways of the DEPs include energy metabolism, amino acid metabolism, translation, folding-sorting degradation, signal transduction, cell motility, cell communication, and immune system (Figure 4A). Weighing the fold changes of proteins in each pathway determined that the weight in the cell motility pathway is the highest (Figure 4A). Moreover, 11 of 20 proteins could participate in this pathway (Figure 4B) via mapping and linking-related pathways. The results strongly suggest that cell motility, including migration, invasion, and adhesion, may be markedly different between UC-MSCs and BM-MSCs.

### Conformation of the differential expression of five proteins

**Figure 5:**
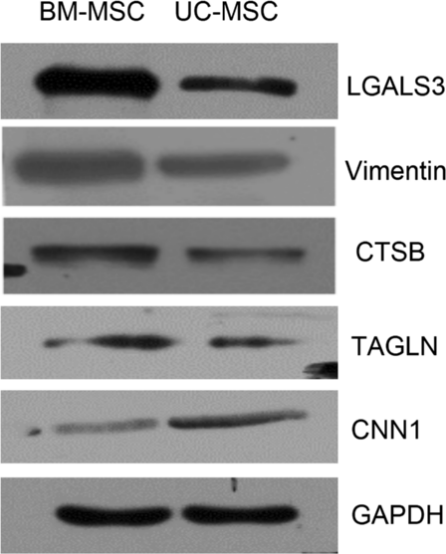
Confirmation of different expression of five proteins. The relative expression levels of five proteins—Vimentin, LGALS3, CTSB, TAGLN, and CNN1—in bone marrow mesenchymal stem cells (BM-MSCs) and umbilical cord mesenchymal stem cells (UC-MSCs) were confirmed with proteomic results.

Significantly DEPs are most likely to affect cellular biological behavior. To verify the quantitative proteomic results, we chose Vimentin and LGALS3 because they exhibited large differences in expression between the two types of MSCs, whereas CTSB, TAGLN, and CNN1 were randomly selected for Western blotting verification (Figure 5). The Western blot results are in agreement with the iTRAQ results. Therefore, the results of the Western blotting analysis confirmed the reliability of the proteomic analysis.

### The cell migration ability of BM-MSCs is higher than that of UC-MSCs

**Figure 6:**
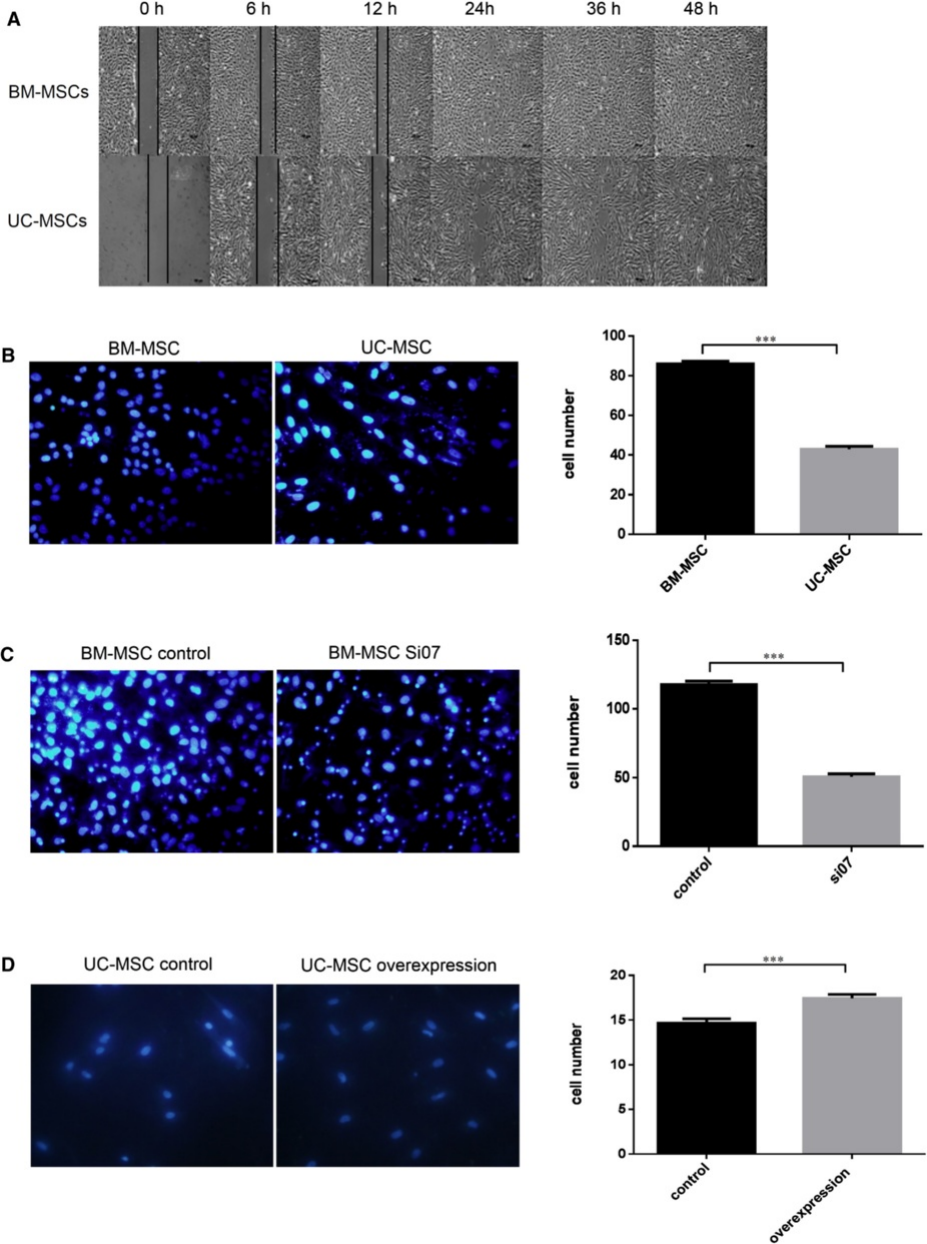
The cell migration ability of bone marrow mesenchymal stem cells (BM-MSCs) was higher than that of umbilical cord mesenchymal stem cells (UC-MSCs), which might be related to Vimentin. **(A)** Zero, six, 12, 24, 36, and 48 hours after scratching in BM-MSCs and UC-MSCs, respectively. Downward lines were drawn to show the healing rate of each scratching even. **(B)** Transwell results of wild-type BM-MSCs and UC-MSCs. **(C, D)** The effect of Vimentin expression on the migration of BM-MSCs and UC-MSCs. The expression of Vimentin was reduced in BM-MSCs (C) and elevated in UC-MSCs (D). As the histograms showed, the migration effect of BM-MSCs is higher than that of UC-MSCs and positively correlated with the expression of Vimentin. ***P <0.01. Bars show the standard error of the mean. Magnifications: 4× (A) and 40× (B-D).

The proteomic data displayed many DEPs related to cell migration, such as Vimentin, LGALS3, FSCN1, TAGLN, and CTSB (Table 1). Scratch and transwell assays were used to detect the migration ability of MSCs. The results of the migration assay showed that BM-MSCs had a greater migration ability than UC-MSCs (Figure 6A, B).

### Vimentin protein positively modulates mesenchymal stem cell migration

Additionally, we found that the expression of the *vimentin* gene is higher in BM-MSCs than in UC-MSCs (Table 1). The Vimentin protein has been positively related to the high metastasis of tumor cells and lymphocytes. Therefore, *vimentin* may play an important role in MSC migration. To test this function, we knocked down *vimentin* in BM-MSCs and overexpressed this gene in UC-MSCs to assess its effect on cell migration (Additional file 1: Figure S1). As shown in Figure 6C and D, the knockdown of *vimentin* in BM-MSCs limits their migration ability; however, *vimentin* overexpression in UC-MSCs promotes migration. These results suggested that the higher migration capability of BM-MSCs may be due to the higher expression of migration-related protein than UC-MSCs.

## Discussion

In recent years, adult MSCs have been widely used in regenerative medicine research, and these cells can be isolated from various tissues. Two types of MSCs were used in this study: UC-MSCs and BM-MSCs. These cells have similar biological characteristics and immunophenotypes. Because UC-MSCs have the advantages of both convenience and ease of cell isolation, they may be used as an alternative therapy candidate over BM-MSCs, which are more difficult to isolate [2]. In addition, comparative proteomic studies indicate that UC-MSCs have a high degree of overlap in protein expression profiles with MSCs compared with embryonic stem cells [4]. UC-MSCs also display differences in their osteogenic differentiation phenotypes. For example, UC-MSCs show few Alizarin Red-positive deposits compared with BM-MSCs; however, the associated mechanism is unclear [17]. Interestingly, comparative proteomic analyses of porcine BM-MSCs and UC-MSCs have not been reported. Therefore, analyzing the DEPs between these two sets of porcine MSCs may reveal molecular mechanisms underlying the self-renewal, differentiation, and tissue homing capacity of these cells and may provide a theoretical basis for the clinical application of MSCs in cell therapy and regenerative medicine [1].

During cell therapy, the integration of the donor cells or biological material and the damaged tissue is dependent on cell migration, differentiation, and matrix remodeling [2,17,18]. In this study, BM-MSCs and UC-MSCs were isolated from newborn piglets, and their cell surface marker molecules, such as CD29, CD31, CD34, CD44, CD45, and CD90, were identified during osteogenic and adipogenic differentiation assays to determine the *in vitro* cell homozygosity of MSCs and to maintain pluripotency.

In this study, we used iTRAQ labeling coupled with LC-MS/MS to conduct a quantitative analysis of DEPs from porcine BM-MSCs and UC-MSCs. This method is not only gel-free but also sensitive and can detect many low-abundance proteins often missed by other proteomic methods. The results showed that 95 DEPs have at least a 39% difference in expression levels in two technical replicates. The DEPs include five proteins that are expressed 10 times more highly in BM-MSCs: LGALS3, VIM, TMSB4X, CALD1, and P4HA1. Three of these proteins were reviewed porcine proteins in the Swiss-Prot protein database, LGALS3, VIM, and TMSB4X, and two were automatically annotated, CALD1 and P4HA1. These proteins may play important roles in the process of cell biology.

We subjected 20 of the DEPs to KEGG pathway analysis and identified several pathways involved in many aspects of cell biology, including metabolism, genetic information processing, environmental information processing, cellular processes, and organismal systems. The most highly represented pathway for the DEPs is the cell motility pathway. Protein detection methods used in our experiment could analyze only the expression level of each protein between different MSCs. But variations in the level of phosphorylation of proteins would not be detected. And in different types of cells, effects of protein phosphorylation on pathway could hardly be compared. However, we attempted to detect the phosphorylation of AKt and Erk in MSCs which showed that phosphorylation of both proteins increased significantly in the BM-MSCs (Additional file 1: Figure S2).

Moreover, we observed that some structural proteins, which are important for structural support of the cell and maintaining the shape of the cell, were differentially expressed in these MSCs. Compared with UC-MSCs, VIM is upregulated, whereas CNN1, MYO1D, and PLS3 are downregulated in BM-MSCs. It has been shown that the expression of CNN1 in smooth muscle increased after transforming growth factor-beta (TGFβ) induced BM-MSC differentiation [19]. In this study, the expression of CNN1 was downregulated in a time-dependent manner (data not published) during the osteogenic differentiation of BM-MSCs. Interestingly, the proteomic results showed that the CNN1 expression level in BM-MSCs was half that of UC-MSCs. Moreover, the osteogenic differentiation experiment suggested that the differentiation ability of BM-MSCs is greater than that of UC-MSCs. Taken together, these results suggest that CNN1 may play an inhibitory role in the osteogenic differentiation of MSCs. These results suggest that the BM-MSCs and UC-MSCs may have some differences in cell motility, including migration, invasion, and adhesion.

In these experiments, the pathway analysis was performed by weighting the analysis with protein abundance data to achieve a better understanding of the differences present in these MSCs. This methodology, compared with analysis methods that consider only the amount of proteins, more comprehensively highlight the different pathways activated in these cells.

Related reports regarding the proteomic differences between human BM-MSCs and UC-MSCs have been published. These studies showed that the expression of proteins that are positive regulators of cell migration, such as LGALS3, VIM, and TMSB4X, are significantly higher in BM-MSCs compared with UC-MSCs and that the expression levels of negative regulators are significantly reduced. Meanwhile, cell migration assays revealed a higher migration ability of BM-MSCs. The relationship between VIM and MSC migration is not well understood. Therefore, we compared the role of VIM in the migration phenotype of porcine BM-MSCs and UC-MSCs. Vimentin was identified in these experiments to be positively related in MSC migration. This type III intermediate filament protein is abundantly expressed in MSCs and in endothelial cells [20]. Although Vimentin is a basic regulatory protein involved in many physiological processes, such as intracellular homeostasis, cell viability, endothelial integrity, and nervous system injury, *vimentin* gene knockout mice showed no obvious barriers to embryonic development and had no effect on the survival of individuals [20,21]. To explore the role of the Vimentin protein in MSC migration, the expression of this protein was knocked down in BM-MSCs and overexpressed in UC-MSCs in our study. Scratch injury and transwell migration tests showed that the migration ability of BM-MSCs was significantly higher than that of UC-MSCs, and this ability is associated with the up-regulated expression of the Vimentin protein. In addition, Rogel MR *et al*. reported that, in alveolar epithelial cells, TGFβ1 could enhance the expression of Vimentin [22]. However, in our proteomic data, we did not find any difference in expression of TGFβ1 between BM-MSCs and UC-MSCs. When the *vimentin* gene was deleted, the expression of adhesion molecules was impaired, thereby reducing the adhesion and trans-endothelial migration rate of lymphocyte cells [23]. A close relation between Vimentin and cancer cell migration capabilities has also been found [24-26]. LGALS3, which is the third member of the galactosidase lectin protein family, was significantly increased in BM-MSCs, and this protein plays an important role in cell proliferation, adhesion, migration, and apoptosis [27-32].

The expression of TMSB4X, a major G-actin-sequestering peptide that is largely distributed in various cells and in the circulatory system and that is capable of binding to G-actin to inhibit actin aggregation, was significantly more highly expressed in BM-MSCs than in UC-MSCs [33,34]. TMSB4X promoted increased expression of MMP-1, MMP-2, and MMP-3 as well as the secretion of plasminogen activator inhibitor-1 (PAI-1), thereby increasing the levels of cell adhesion and migration [35]. The regulation of cell migration by TMSB4X is cell-specific. In endothelial cells, the transcription level of PAI-1 is enhanced by TMSB4X [35]. However, comparative proteomic studies of BM-MSCs and UC-MSCs showed that the expression of PAI-1 in BM-MSCs was significantly lower than that in UC-MSCs, and the cell migration assay results indicated that PAI-1 is a negative regulator of cell migration in MSCs [2]. Thus, the effect of TMSB4X on endothelial cell migration is most likely regulated by a complex variety of factors. In tumor cells, TMSB4X can promote cell migration and vascular endothelial growth factor-induced angiogenesis, thereby accelerating tumor growth and metastasis [36,37]. Lastly, TMSB4X is an anti-inflammatory factor that could accelerate the repair of skin and corneal burns, a process which is closely related to cell migration promoted by TMSB4X [38].

One interesting result of this study is that proteins related to the immune response, such as PRKCSH, Oas2, and RIG-I, are differentially expressed between BM-MSCs and UC-MSCs. PRKCSH is a regulatory subunit of glucosidase II and is expressed predominantly in the endoplasmic reticulum [39]. In fetal tissue, PRKCSH is expressed in the ductal plate, bile ducts, and hepatocytes, suggesting that PRKCSH is most likely required for bile duct development [39]. RIG-I is a member of the RIG-I-like receptor family and plays a major role in pathogen sensing of RNA virus infection to induce type I interferon production [40]. However, strong evidence has shown that significant induction of RIG-I occurs during normal myelopoiesis and that the development of a progressive myeloproliferative disorder is disrupted when Rig-I is knocked down [41].

## Conclusions

This study is the first of its kind to perform a quantitative analysis of DEPs between porcine BM-MSCs and UC-MSCs, combined with GO analysis, KEGG signaling pathway analysis, and cell migration studies. These experiments describe differences in the migration abilities of these two types of cells. The data indicate that this difference in migration is related to the expression levels of proteins, and therefore, they provide experimental evidence for cell-based targeted therapy using MSCs.

## Additional file

Additional file 1:
**Table S1.** Gene Ontology annotations of differentially expressed proteins. **Table S2.** Kyoto Encyclopedia of Genes and Genomes (KEGG) pathways of differentially expressed proteins. **Table S3.** Primer information. **Figure S1.** Results of real-time polymerase chain reaction (PCR) and Western blotting of mesenchymal stem cells (MSCs). **Figure S2.** Phosphorylation level detection of AKt and Erk in mesenchymal stem cells (MSCs). **Figure S3.** Histograms of immunophenotypic profiling of bone marrow mesenchymal stem cells (BM-MSCs). **Figure S4.** Histograms of immunophenotypic profiling of umbilical cord mesenchymal stem cells (UC-MSCs).

## Abbreviations

BM-MSC: bone marrow mesenchymal stem cell
DEP: differentially expressed protein
DMEM: Dulbecco’s modified Eagle’s medium
FBS: fetal bovine serum
GO: Gene Ontology
HPLC: high-performance liquid chromatography
iTRAQ: isobaric tag for relative and absolute quantitation
KEGG: Kyoto Encyclopedia of Genes and Genomes
LC-MS/MS: liquid chromatography-tandem mass spectrometry
MS: mass spectrometry
MSC: mesenchymal stem cell
MS/MS: tandem mass spectrometry
PAI-1: plasminogen activator inhibitor-1
PBS: phosphate-buffered saline
PSI: pounds per square inch
SCX: strong cation exchange
siRNA: short interfering RNA
TGFβ1: transforming growth factor-beta
TOF: time-of-flight
UC-MSC: umbilical cord mesenchymal stem cell
