# Supplementary material for: Proteomic analysis of porcine mesenchymal stem cells derived from bone marrow and umbilical cord: implication of the proteins involved in the higher migration capability of bone marrow mesenchymal stem cells
Source: Stem Cell Res Ther. 2015 Apr 15;6(1):77. doi: 10.1186/s13287-015-0061-x (PMC4425931; doi:10.1186/s13287-015-0061-x)
Supplement: Additional file 1: — Table S1. Gene Ontology annotations of differentially expressed proteins. Table S2. Kyoto Encyclopedia of Genes and Genomes (KEGG) pathways of differentially expressed proteins. Table S3. Primer information. Figure S1. Results of real-time polymerase chain reaction (PCR) and Western blotting of mesenchymal stem cells (MSCs). Figure S2. Phosphorylation level detection of AKt and Erk in mesenchymal stem cells (MSCs). Figure S3. Histograms of immunophenotypic profiling of bone marrow mesenchymal stem cells (BM-MSCs). Figure S4. Histograms of immunophenotypic profiling of umbilical cord mesenchymal stem cells (UC-MSCs). [file 13287_2015_61_MOESM1_ESM.pdf]

Table S1 Gene Ontology Annotations of Differentially Expressed Proteins.

Table S2 KEGG pathways of differentially expressed proteins.

Table S3 Primers information.

Figure S1 Results of Real-Time PCR and western-blotting of MSCs.

Figure S2 Phosphorylation level detection of AKt and Erk in MSCs.

Figure S3 Histograms of immunophenotypic profiling of BM-MSCs.

Figure S4 Histograms of immunophenotypic profiling of UC-MSCs.

Figure legends

Figure S1 Results of Real-Time PCR and western-blotting of MSCs. SiRNAs named si07, si09 and si11 were used to interfere the translation of vimentin. According to the RQ results of real - time PCR, si07 was the most useful siRNA which was chose for cell experiments. At the same time, Viemntin protein was overexpressed and the protein amount was tested in western - blotting. ***, $p < 0.01$. Bars showed the SEM. +, overexpression.

Figure S2 Phosphorylation level detection of AKt and Erk in MSCs. Western blotting results showed that the phosphorylation levels of AKt and Erk in BM-MSCs were higher than which in UC-MSCs.

Figure S3 Histograms of immunophenotypic profiling of BM-MSCs. Flow cytometric analysis of cell surface markers of BM-MSCs. BM-MSCs expressed CD29, CD44 and CD90 but not CD34 and CD45.

Figure S4 Histograms of immunophenotypic profiling of UC-MSCs. Flow cytometric analysis of cell surface markers of UC-MSCs. UC-MSCs expressed CD90 but not CD31, CD34 and CD45.

Table S1 Gene Ontology Annotations of Differentially Expressed Proteins

Uniprot_AC	GO_Identifier	GO_Name	Aspect
**A3EX84**	GO:0030198	extracellular matrix organization	BP
**A3EX84**	GO:0001501	skeletal system development	BP
**P02543**	GO:0014002	astrocyte development	BP
**P02543**	GO:0060020	Bergmann glial cell differentiation	BP
**P02543**	GO:0045109	intermediate filament organization	BP
**P02543**	GO:0070307	lens fiber cell development	BP
**P02543**	GO:0010977	negative regulation of neuron projection development	BP
**B3XXC3**	GO:0030036	actin cytoskeleton organization	BP
**B3XXC3**	GO:0042989	sequestering of actin monomers	BP
**F1SNH3**	GO:0006936	muscle contraction	BP
**A1X898**	GO:0030199	collagen fibril organization	BP
**A1X898**	GO:0018401	peptidyl-proline hydroxylation to 4-hydroxy-L-proline	BP
**F1SU97**	GO:0006665	sphingolipid metabolic process	BP
**F1SKI0**	GO:0048739	cardiac muscle fiber development	BP
**F1SKI0**	GO:0048251	elastic fiber assembly	BP
**F1SKI0**	GO:0006939	smooth muscle contraction	BP
**F1SK03**	GO:0006508	proteolysis	BP
**Q56VQ1**	GO:0051607	defense response to virus	BP
**Q56VQ1**	GO:0006955	immune response	BP
**F1SEQ7**	GO:0055114	oxidation-reduction process	BP
**F1SEQ7**	GO:0045670	regulation of osteoclast differentiation	BP
**F2Z5M2**	GO:0071229	cellular response to acid	BP
**F2Z5M2**	GO:0051099	positive regulation of binding	BP
**Q2YGT9**	GO:0006412	translation	BP
**F1RQW2**	GO:0010951	negative regulation of endopeptidase activity	BP
**F1RKW9**	GO:0010923	negative regulation of phosphatase activity	BP
**F1SDX6**	GO:0043277	apoptotic cell clearance	BP
**F1SDX6**	GO:0060445	branching involved in salivary gland morphogenesis	BP
**F1SDX6**	GO:0018149	peptide cross-linking	BP
**F1SDX6**	GO:0045785	positive regulation of cell adhesion	BP
**F1SDX6**	GO:0060662	salivary gland cavitation	BP
**F1S663**	GO:0016477	cell migration	BP
**F1S663**	GO:0022617	extracellular matrix disassembly	BP
**F1S663**	GO:0031581	hemidesmosome assembly	BP
**F1S663**	GO:0031175	neuron projection development	BP
**F1S663**	GO:0006461	protein complex assembly	BP
**F1S663**	GO:0034446	substrate adhesion-dependent cell spreading	BP
**F1SKJ1**	GO:0031532	actin cytoskeleton reorganization	BP
**F1SKJ1**	GO:0030048	actin filament-based movement	BP
**F1SKJ1**	GO:0001525	angiogenesis	BP
**F1SKJ1**	GO:0043534	blood vessel endothelial cell migration	BP
**F1SKJ1**	GO:0016337	cell-cell adhesion	BP
**F1SKJ1**	GO:0000910	cytokinesis	BP
**F1SKJ1**	GO:0051295	establishment of meiotic spindle localization	BP
**F1SKJ1**	GO:0001768	establishment of T cell polarity	BP
**F1SKJ1**	GO:0001701	in utero embryonic development	BP
**F1SKJ1**	GO:0000212	meiotic spindle organization	BP
**F1SKJ1**	GO:0006509	membrane protein ectodomain proteolysis	BP
**F1SKJ1**	GO:0030224	monocyte differentiation	BP
**F1SKJ1**	GO:0007520	myoblast fusion	BP
**F1SKJ1**	GO:0030220	platelet formation	BP

Uniprot_AC	GO_Identifier	GO_Name	Aspect
**F1SKJ1**	GO:0015031	protein transport	BP
**F1SKJ1**	GO:0008360	regulation of cell shape	BP
**F1SKJ1**	GO:0032796	uropod organization	BP
**Q9TSX9**	GO:0009395	phospholipid catabolic process	BP
**Q9TSX9**	GO:0006979	response to oxidative stress	BP
**F1RGS2**	GO:0005975	carbohydrate metabolic process	BP
**F1RGS2**	GO:0071356	cellular response to tumor necrosis factor	BP
**F1RGS2**	GO:0046513	ceramide biosynthetic process	BP
**F1RGS2**	GO:0006680	glucosylceramide catabolic process	BP
**F1RGS2**	GO:0032715	negative regulation of interleukin-6 production	BP
**F1RGS2**	GO:0043407	negative regulation of MAP kinase activity	BP
**F1RGS2**	GO:0035307	positive regulation of protein dephosphorylation	BP
**F1RGS2**	GO:0046512	sphingosine biosynthetic process	BP
**F1RGS2**	GO:0023021	termination of signal transduction	BP
**F1SLI3**	GO:0051301	cell division	BP
**F1SLI3**	GO:0051294	establishment of spindle orientation	BP
**F1SLI3**	GO:0051012	microtubule sliding	BP
**F1SLI3**	GO:0007052	mitotic spindle organization	BP
**C6K7I0**	GO:0006606	protein import into nucleus	BP
**P20112**	GO:0007165	signal transduction	BP
**P27594**	GO:0051607	defense response to virus	BP
**P27594**	GO:0006184	GTP catabolic process	BP
**P27594**	GO:0045087	innate immune response	BP
**F1S4Y8**	GO:0001701	in utero embryonic development	BP
**F1S4Y8**	GO:0002176	male germ cell proliferation	BP
**F1S4Y8**	GO:0008584	male gonad development	BP
**F1RSC3**	GO:0006508	proteolysis	BP
**F1SAP4**	GO:0045765	regulation of angiogenesis	BP
**F1SAP4**	GO:0006436	tryptophanyl-tRNA aminoacylation	BP
**Q08092**	GO:0031032	actomyosin structure organization	BP
**F1RST0**	GO:0051085	chaperone mediated protein folding requiring cofactor	BP
**F2Z5K2**	GO:0006511	ubiquitin-dependent protein catabolic process	BP
**F1RRV6**	GO:0071456	cellular response to hypoxia	BP
**F1RRV6**	GO:0030330	DNA damage response, signal transduction by p53 class mediator	BP
**F1RRV6**	GO:0045576	mast cell activation	BP
**F1RRV6**	GO:0032287	peripheral nervous system myelin maintenance	BP
**F1RRV6**	GO:0090232	positive regulation of spindle checkpoint	BP
**F1SLA0**	GO:0001525	angiogenesis	BP
**F1SLA0**	GO:0015991	ATP hydrolysis coupled proton transport	BP
**F1SLA0**	GO:0015986	ATP synthesis coupled proton transport	BP
**F1SLA0**	GO:0006629	lipid metabolic process	BP
**F1SLA0**	GO:0006933	negative regulation of cell adhesion involved in substrate-bound cell migration	BP
**F1SLA0**	GO:0051453	regulation of intracellular pH	BP
**F1SQ11**	GO:0006897	endocytosis	BP
**F1SQ11**	GO:0006906	vesicle fusion	BP
**A5A768**	GO:0006886	intracellular protein transport	BP
**A5A768**	GO:0016192	vesicle-mediated transport	BP
**F1S554**	GO:0008360	regulation of cell shape	BP
**F1SGP8**	GO:0043010	camera-type eye development	BP
**F1SGP8**	GO:0001701	in utero embryonic development	BP
**F1SMN5**	GO:0048747	muscle fiber development	BP
**F1SIJ9**	GO:0006564	L-serine biosynthetic process	BP
**F2Z5C1**	GO:0050819	negative regulation of coagulation	BP

Uniprot_AC	GO_Identifier	GO_Name	Aspect
**F2Z5C1**	GO:0010033	response to organic substance	BP
**B6CVD6**	GO:0045454	cell redox homeostasis	BP
**F1RI39**	GO:0051271	negative regulation of cellular component movement	BP
**F1RI39**	GO:0051272	positive regulation of cellular component movement	BP
**F1RI39**	GO:0048549	positive regulation of pinocytosis	BP
**F1SJJ5**	GO:0006412	translation	BP
**F1RJ93**	GO:0007517	muscle organ development	BP
**F1SS24**	GO:0007161	calcium-independent cell-matrix adhesion	BP
**F1SS24**	GO:0007044	cell-substrate junction assembly	BP
**F1SS24**	GO:0018149	peptide cross-linking	BP
**F1SS24**	GO:0034446	substrate adhesion-dependent cell spreading	BP
**F1SS24**	GO:0042060	wound healing	BP
**P33198**	GO:0006103	2-oxoglutarate metabolic process	BP
**P33198**	GO:0006097	glyoxylate cycle	BP
**P33198**	GO:0006102	isocitrate metabolic process	BP
**P33198**	GO:0006099	tricarboxylic acid cycle	BP
**F1RWJ5**	GO:0006610	ribosomal protein import into nucleus	BP
**F1S3M9**	GO:0030866	cortical actin cytoskeleton organization	BP
**P28491**	GO:0007050	cell cycle arrest	BP
**P28491**	GO:0090398	cellular senescence	BP
**P28491**	GO:0030866	cortical actin cytoskeleton organization	BP
**P28491**	GO:0033144	negative regulation of intracellular steroid hormone receptor signaling pathway	BP
**P28491**	GO:0045665	negative regulation of neuron differentiation	BP
**P28491**	GO:0048387	negative regulation of retinoic acid receptor signaling pathway	BP
**P28491**	GO:0000122	negative regulation of transcription from RNA polymerase II promoter	BP
**P28491**	GO:0002502	peptide antigen assembly with MHC class I protein complex	BP
**P28491**	GO:0045787	positive regulation of cell cycle	BP
**P28491**	GO:0008284	positive regulation of cell proliferation	BP
**P28491**	GO:2000510	positive regulation of dendritic cell chemotaxis	BP
**P28491**	GO:0045740	positive regulation of DNA replication	BP
**P28491**	GO:0010628	positive regulation of gene expression	BP
**P28491**	GO:0050766	positive regulation of phagocytosis	BP
**P28491**	GO:1900026	positive regulation of substrate adhesion-dependent cell spreading	BP
**P28491**	GO:0006611	protein export from nucleus	BP
**P28491**	GO:0006457	protein folding	BP
**P28491**	GO:0034504	protein localization to nucleus	BP
**P28491**	GO:0050821	protein stabilization	BP
**P28491**	GO:0040020	regulation of meiosis	BP
**Q4GWZ2**	GO:0007155	cell adhesion	BP
**Q4GWZ2**	GO:0009615	response to virus	BP
**Q4GWZ2**	GO:0000028	ribosomal small subunit assembly	BP
**Q4GWZ2**	GO:0006412	translation	BP
**B2CNZ7**	GO:0006508	proteolysis	BP
**B2CNZ7**	GO:0050790	regulation of catalytic activity	BP
**F1SJR7**	GO:0006464	cellular protein modification process	BP
**G9F6X8**	GO:0045454	cell redox homeostasis	BP
**G9F6X8**	GO:0006662	glycerol ether metabolic process	BP
**F1RGP1**	GO:0042149	cellular response to glucose starvation	BP
**F1RGP1**	GO:0072332	intrinsic apoptotic signaling pathway by p53 class mediator	BP
**F1RGP1**	GO:0045892	negative regulation of transcription, DNA-templated	BP
**F1RGP1**	GO:0006913	nucleocytoplasmic transport	BP
**F1RGP1**	GO:0071158	positive regulation of cell cycle arrest	BP
**F1RGP1**	GO:0022904	respiratory electron transport chain	BP

Uniprot_AC	GO_Identifier	GO_Name	Aspect
**F1RGP1**	GO:0006351	transcription, DNA-templated	BP
**Q5MJE5**	GO:0006508	proteolysis	BP
**F1RI15**	GO:0051131	chaperone-mediated protein complex assembly	BP
**F1RI15**	GO:0045040	protein import into mitochondrial outer membrane	BP
**F1SFZ8**	GO:0007155	cell adhesion	BP
**F1SFZ8**	GO:0007044	cell-substrate junction assembly	BP
**F1SFZ8**	GO:0030866	cortical actin cytoskeleton organization	BP
**F1SFZ8**	GO:0007016	cytoskeletal anchoring at plasma membrane	BP
**Q8SPT0**	GO:0006541	glutamine metabolic process	BP
**D0G0C6**	GO:0070981	L-asparagine biosynthetic process	BP
**D0G0C6**	GO:0043066	negative regulation of apoptotic process	BP
**D0G0C6**	GO:0045931	positive regulation of mitotic cell cycle	BP
**F1RS36**	GO:0006987	activation of signaling protein activity involved in unfolded protein response	BP
**F1RS36**	GO:0042149	cellular response to glucose starvation	BP
**F1RS36**	GO:0071353	cellular response to interleukin-4	BP
**F1RS36**	GO:0021680	cerebellar Purkinje cell layer development	BP
**F1RS36**	GO:0021589	cerebellum structural organization	BP
**F1RS36**	GO:0006983	ER overload response	BP
**F1RS36**	GO:0043066	negative regulation of apoptotic process	BP
**F1RS36**	GO:0030512	negative regulation of transforming growth factor beta receptor signaling pathway	BP
**F1RS36**	GO:0031398	positive regulation of protein ubiquitination	BP
**F1RS36**	GO:0051603	proteolysis involved in cellular protein catabolic process	BP
**F1S0V3**	GO:0006816	calcium ion transport	BP
**F1S0V3**	GO:0006937	regulation of muscle contraction	BP
**F1SK12**	GO:0048675	axon extension	BP
**F1SK12**	GO:0016358	dendrite development	BP
**F1SK12**	GO:0061162	establishment of monopolar cell polarity	BP
**F1SK12**	GO:0001578	microtubule bundle formation	BP
**F1SK12**	GO:0047497	mitochondrion transport along microtubule	BP
**F1SK12**	GO:0032387	negative regulation of intracellular transport	BP
**F1SK12**	GO:0045773	positive regulation of axon extension	BP
**F1SL58**	GO:0007017	microtubule-based process	BP
**F1SL58**	GO:0007067	mitosis	BP
**Q59IP2**	GO:0071230	cellular response to amino acid stimulus	BP
**Q59IP2**	GO:0030199	collagen fibril organization	BP
**Q59IP2**	GO:0048592	eye morphogenesis	BP
**Q59IP2**	GO:0001501	skeletal system development	BP
**Q59IP2**	GO:0043588	skin development	BP
**Q29092**	GO:0030433	ER-associated ubiquitin-dependent protein catabolic process	BP
**Q29092**	GO:0006457	protein folding	BP
**Q29092**	GO:0006950	response to stress	BP
**F1S596**	GO:0001701	in utero embryonic development	BP
**F1S596**	GO:0001889	liver development	BP
**F1S596**	GO:0006491	N-glycan processing	BP
**F1S596**	GO:0010977	negative regulation of neuron projection development	BP
**F1S596**	GO:0006807	nitrogen compound metabolic process	BP
**F1S596**	GO:0072001	renal system development	BP
**P80021**	GO:0006754	ATP biosynthetic process	BP
**P80021**	GO:0015991	ATP hydrolysis coupled proton transport	BP
**P80021**	GO:0015986	ATP synthesis coupled proton transport	BP
**P80021**	GO:0009790	embryo development	BP
**P80021**	GO:0006629	lipid metabolic process	BP
**P80021**	GO:0001937	negative regulation of endothelial cell proliferation	BP

Uniprot_AC	GO_Identifier	GO_Name	Aspect
**P80021**	GO:0015992	proton transport	BP
**B0LXK8**	GO:0006166	purine ribonucleoside salvage	BP
**A3EX84**	GO:0005743	mitochondrial inner membrane	CC
**A3EX84**	GO:0005634	nucleus	CC
**A3EX84**	GO:0005578	proteinaceous extracellular matrix	CC
**P02543**	GO:0031252	cell leading edge	CC
**P02543**	GO:0042995	cell projection	CC
**P02543**	GO:0005829	cytosol	CC
**P02543**	GO:0070062	extracellular vesicular exosome	CC
**P02543**	GO:0005882	intermediate filament	CC
**P02543**	GO:0005777	peroxisome	CC
**P02543**	GO:0005886	plasma membrane	CC
**B3XXC3**	GO:0005737	cytoplasm	CC
**A1X898**	GO:0005581	collagen	CC
**A1X898**	GO:0005783	endoplasmic reticulum	CC
**A1X898**	GO:0005739	mitochondrion	CC
**F1SU97**	GO:0005615	extracellular space	CC
**F1SU97**	GO:0005764	lysosome	CC
**F1SKI0**	GO:0005859	muscle myosin complex	CC
**F1SKI0**	GO:0030485	smooth muscle contractile fiber	CC
**F1SKI0**	GO:0001725	stress fiber	CC
**F1SK03**	GO:0005793	endoplasmic reticulum-Golgi intermediate compartment	CC
**F1SK03**	GO:0009897	external side of plasma membrane	CC
**F1SEQ7**	GO:0005739	mitochondrion	CC
**F2Z5M2**	GO:0019897	extrinsic component of plasma membrane	CC
**F1RWW4**	GO:0030018	Z disc	CC
**Q2YGT9**	GO:0022625	cytosolic large ribosomal subunit	CC
**D0G7F7**	GO:0030863	cortical cytoskeleton	CC
**D0G7F7**	GO:0031941	filamentous actin	CC
**D0G7F7**	GO:0002102	podosome	CC
**D0G7F7**	GO:0001725	stress fiber	CC
**F1RQW2**	GO:0005615	extracellular space	CC
**A9YUA9**	GO:0005783	endoplasmic reticulum	CC
**F1RKW9**	GO:0016459	myosin complex	CC
**F1S663**	GO:0070062	extracellular vesicular exosome	CC
**F1S663**	GO:0043259	laminin-10 complex	CC
**F1SKJ1**	GO:0005826	actomyosin contractile ring	CC
**F1SKJ1**	GO:0005913	cell-cell adherens junction	CC
**F1SKJ1**	GO:0032154	cleavage furrow	CC
**F1SKJ1**	GO:0008180	COP9 signalosome	CC
**F1SKJ1**	GO:0030863	cortical cytoskeleton	CC
**F1SKJ1**	GO:0005829	cytosol	CC
**F1SKJ1**	GO:0070062	extracellular vesicular exosome	CC
**F1SKJ1**	GO:0001772	immunological synapse	CC
**F1SKJ1**	GO:0008305	integrin complex	CC
**F1SKJ1**	GO:0016460	myosin II complex	CC
**F1SKJ1**	GO:0031594	neuromuscular junction	CC
**F1SKJ1**	GO:0001726	ruffle	CC
**F1SKJ1**	GO:0005819	spindle	CC
**F1SKJ1**	GO:0001725	stress fiber	CC
**F1SKJ1**	GO:0001931	uropod	CC
**Q9TSX9**	GO:0005737	cytoplasm	CC
**Q9TSX9**	GO:0016023	cytoplasmic membrane-bounded vesicle	CC

Uniprot_AC	GO_Identifier	GO_Name	Aspect
**Q9TSX9**	GO:0005764	lysosome	CC
**F1RGS2**	GO:0043202	lysosomal lumen	CC
**F1RGS2**	GO:0005765	lysosomal membrane	CC
**F1SLI3**	GO:0005737	cytoplasm	CC
**F1SLI3**	GO:0005874	microtubule	CC
**F1SLI3**	GO:0072686	mitotic spindle	CC
**F1SLI3**	GO:0005886	plasma membrane	CC
**F1SMN1**	GO:0005783	endoplasmic reticulum	CC
**C6K7I0**	GO:0005737	cytoplasm	CC
**C6K7I0**	GO:0005634	nucleus	CC
**P20112**	GO:0005604	basement membrane	CC
**P20112**	GO:0005615	extracellular space	CC
**P27594**	GO:0005737	cytoplasm	CC
**P27594**	GO:0005789	endoplasmic reticulum membrane	CC
**P27594**	GO:0048471	perinuclear region of cytoplasm	CC
**C0LZL0**	GO:0005737	cytoplasm	CC
**C0LZL0**	GO:0005856	cytoskeleton	CC
**F1RJL6**	GO:0005881	cytoplasmic microtubule	CC
**F1SAP4**	GO:0005737	cytoplasm	CC
**Q08092**	GO:0005856	cytoskeleton	CC
**F1RST0**	GO:0005737	cytoplasm	CC
**F1RST0**	GO:0005874	microtubule	CC
**F1RST0**	GO:0005634	nucleus	CC
**F2Z5K2**	GO:0005737	cytoplasm	CC
**F2Z5K2**	GO:0005634	nucleus	CC
**F2Z5K2**	GO:0019773	proteasome core complex, alpha-subunit complex	CC
**F1RRV6**	GO:0005913	cell-cell adherens junction	CC
**F1RRV6**	GO:0005813	centrosome	CC
**F1RRV6**	GO:0005874	microtubule	CC
**F1RRV6**	GO:0005634	nucleus	CC
**F1RRV6**	GO:0048471	perinuclear region of cytoplasm	CC
**F1RRV6**	GO:0005886	plasma membrane	CC
**F1RRV6**	GO:0055038	recycling endosome membrane	CC
**F1SLA0**	GO:0009986	cell surface	CC
**F1SLA0**	GO:0042645	mitochondrial nucleoid	CC
**F1SLA0**	GO:0005753	mitochondrial proton-transporting ATP synthase complex	CC
**F1SLA0**	GO:0005886	plasma membrane	CC
**F1SLA0**	GO:0045261	proton-transporting ATP synthase complex, catalytic core F(1)	CC
**F1SQ11**	GO:0005829	cytosol	CC
**F1SQ11**	GO:0005769	early endosome	CC
**F1SQ11**	GO:0019897	extrinsic component of plasma membrane	CC
**F1SQ11**	GO:0005969	serine-pyruvate aminotransferase complex	CC
**F1S764**	GO:0005739	mitochondrion	CC
**F1S764**	GO:0005730	nucleolus	CC
**A5A768**	GO:0005794	Golgi apparatus	CC
**A5A768**	GO:0030117	membrane coat	CC
**F1S554**	GO:0005737	cytoplasm	CC
**F1S554**	GO:0016020	membrane	CC
**F1SGP8**	GO:0005783	endoplasmic reticulum	CC
**F1SMN5**	GO:0005737	cytoplasm	CC
**F1SMN5**	GO:0042383	sarcolemma	CC
**F1S827**	GO:0005737	cytoplasm	CC
**F1SV06**	GO:0010494	cytoplasmic stress granule	CC

Uniprot_AC	GO_Identifier	GO_Name	Aspect
**F1SV06**	GO:0005634	nucleus	CC
**F1S9A4**	GO:0005783	endoplasmic reticulum	CC
**F1S9A4**	GO:0005793	endoplasmic reticulum-Golgi intermediate compartment	CC
**F1S9A4**	GO:0005794	Golgi apparatus	CC
**F1S9A4**	GO:0005640	nuclear outer membrane	CC
**F2Z5C1**	GO:0072563	endothelial microparticle	CC
**F2Z5C1**	GO:0009897	external side of plasma membrane	CC
**F2Z5C1**	GO:0070062	extracellular vesicular exosome	CC
**F2Z5C1**	GO:0005622	intracellular	CC
**F1RI39**	GO:0030863	cortical cytoskeleton	CC
**F1RI39**	GO:0070062	extracellular vesicular exosome	CC
**F1RI39**	GO:0005634	nucleus	CC
**F1RI39**	GO:0048471	perinuclear region of cytoplasm	CC
**F1RI39**	GO:0043234	protein complex	CC
**F1RI39**	GO:0031143	pseudopodium	CC
**F1RI39**	GO:0030529	ribonucleoprotein complex	CC
**F1RI39**	GO:0001725	stress fiber	CC
**F1RI39**	GO:0030018	Z disc	CC
**F1SPP8**	GO:0005783	endoplasmic reticulum	CC
**F1SPP8**	GO:0005811	lipid particle	CC
**F1SJJ5**	GO:0022625	cytosolic large ribosomal subunit	CC
**F1SJJ5**	GO:0005730	nucleolus	CC
**F1SS24**	GO:0016324	apical plasma membrane	CC
**F1SS24**	GO:0005604	basement membrane	CC
**F1SS24**	GO:0005793	endoplasmic reticulum-Golgi intermediate compartment	CC
**F1SS24**	GO:0005577	fibrinogen complex	CC
**F1SGJ6**	GO:0016021	integral component of membrane	CC
**P33198**	GO:0005743	mitochondrial inner membrane	CC
**F1RWJ5**	GO:0005737	cytoplasm	CC
**F1RWJ5**	GO:0031965	nuclear membrane	CC
**F1RWJ5**	GO:0043234	protein complex	CC
**F1S3M9**	GO:0005737	cytoplasm	CC
**F1S3M9**	GO:0005856	cytoskeleton	CC
**F1S3M9**	GO:0019898	extrinsic component of membrane	CC
**F1SSA6**	GO:0016459	myosin complex	CC
**B0LY42**	GO:0016020	membrane	CC
**P28491**	GO:0005829	cytosol	CC
**P28491**	GO:0009897	external side of plasma membrane	CC
**P28491**	GO:0005615	extracellular space	CC
**P28491**	GO:0042824	MHC class I peptide loading complex	CC
**P28491**	GO:0048471	perinuclear region of cytoplasm	CC
**P28491**	GO:0005844	polysome	CC
**P28491**	GO:0033018	sarcoplasmic reticulum lumen	CC
**Q4GWZ2**	GO:0022627	cytosolic small ribosomal subunit	CC
**Q4GWZ2**	GO:0005634	nucleus	CC
**Q4GWZ2**	GO:0005886	plasma membrane	CC
**G9F6X8**	GO:0005783	endoplasmic reticulum	CC
**F1RWT2**	GO:0005737	cytoplasm	CC
**F1RGP1**	GO:0005737	cytoplasm	CC
**F1RGP1**	GO:0042564	NLS-dependent protein nuclear import complex	CC
**F1RGP1**	GO:0005730	nucleolus	CC
**F1RI15**	GO:0005634	nucleus	CC
**F1SFZ8**	GO:0015629	actin cytoskeleton	CC

Uniprot_AC	GO_Identifier	GO_Name	Aspect
**F1SFZ8**	GO:0005813	centrosome	CC
**F1SFZ8**	GO:0070062	extracellular vesicular exosome	CC
**F1SFZ8**	GO:0005925	focal adhesion	CC
**F1SFZ8**	GO:0043231	intracellular membrane-bounded organelle	CC
**F1SFZ8**	GO:0005886	plasma membrane	CC
**F1SFZ8**	GO:0001726	ruffle	CC
**F1RS36**	GO:0009986	cell surface	CC
**F1RS36**	GO:0008180	COP9 signalosome	CC
**F1RS36**	GO:0034663	endoplasmic reticulum chaperone complex	CC
**F1RS36**	GO:0005793	endoplasmic reticulum-Golgi intermediate compartment	CC
**F1RS36**	GO:0030176	integral component of endoplasmic reticulum membrane	CC
**F1RS36**	GO:0030496	midbody	CC
**F1S0V3**	GO:0070062	extracellular vesicular exosome	CC
**F1S0V3**	GO:0048471	perinuclear region of cytoplasm	CC
**F1SK12**	GO:0005829	cytosol	CC
**F1SK12**	GO:0005874	microtubule	CC
**F1SK12**	GO:0005886	plasma membrane	CC
**F1SL58**	GO:0005869	dynactin complex	CC
**Q59IP2**	GO:0005588	collagen type V	CC
**Q29092**	GO:0005788	endoplasmic reticulum lumen	CC
**Q29092**	GO:0042470	melanosome	CC
**P80021**	GO:0008180	COP9 signalosome	CC
**P80021**	GO:0005743	mitochondrial inner membrane	CC
**P80021**	GO:0005753	mitochondrial proton-transporting ATP synthase complex	CC
**P80021**	GO:0005886	plasma membrane	CC
**P80021**	GO:0045261	proton-transporting ATP synthase complex, catalytic core F(1)	CC
**F1RR78**	GO:0032437	cuticular plate	CC
**F1RR78**	GO:0005916	fascia adherens	CC
**F1RR78**	GO:0043231	intracellular membrane-bounded organelle	CC
**F1RR78**	GO:0016328	lateral plasma membrane	CC
**F1RR78**	GO:0015630	microtubule cytoskeleton	CC
**F1RR78**	GO:0030018	Z disc	CC
**B0LXK8**	GO:0005737	cytoplasm	CC
**A3EX84**	GO:0030246	carbohydrate binding	MF
**P02543**	GO:0005200	structural constituent of cytoskeleton	MF
**P02543**	GO:0005212	structural constituent of eye lens	MF
**A1X898**	GO:0005506	iron ion binding	MF
**A1X898**	GO:0031418	L-ascorbic acid binding	MF
**A1X898**	GO:0016702	oxidoreductase activity, acting on single donors with incorporation of molecular oxygen,	MF
**A1X898**	GO:0004656	procollagen-proline 4-dioxygenase activity	MF
**F1SKI0**	GO:0005524	ATP binding	MF
**F1SKI0**	GO:0003774	motor activity	MF
**F1SKI0**	GO:0008307	structural constituent of muscle	MF
**F1SK03**	GO:0008237	metallopeptidase activity	MF
**F1SK03**	GO:0008270	zinc ion binding	MF
**Q56VQ1**	GO:0005524	ATP binding	MF
**Q56VQ1**	GO:0003725	double-stranded RNA binding	MF
**Q56VQ1**	GO:0016779	nucleotidyltransferase activity	MF
**F1SEQ7**	GO:0016209	antioxidant activity	MF
**F2Z5M2**	GO:0005509	calcium ion binding	MF
**F1RWW4**	GO:0008270	zinc ion binding	MF
**Q2YGT9**	GO:0003735	structural constituent of ribosome	MF
**F1RQW2**	GO:0004866	endopeptidase inhibitor activity	MF

Uniprot_AC	GO_Identifier	GO_Name	Aspect
**A9YUA9**	GO:0008201	heparin binding	MF
**F1RKW9**	GO:0005524	ATP binding	MF
**F1RKW9**	GO:0003774	motor activity	MF
**F1SDX6**	GO:0046872	metal ion binding	MF
**F1SDX6**	GO:0003810	protein-glutamine gamma-glutamyltransferase activity	MF
**F1S663**	GO:0005201	extracellular matrix structural constituent	MF
**F1S663**	GO:0043208	glycosphingolipid binding	MF
**F1SKJ1**	GO:0030898	actin-dependent ATPase activity	MF
**F1SKJ1**	GO:0043531	ADP binding	MF
**F1SKJ1**	GO:0005524	ATP binding	MF
**F1SKJ1**	GO:0000146	microfilament motor activity	MF
**Q9TSX9**	GO:0004602	glutathione peroxidase activity	MF
**Q9TSX9**	GO:0051920	peroxiredoxin activity	MF
**Q9TSX9**	GO:0004623	phospholipase A2 activity	MF
**F1RGS2**	GO:0004348	glucosylceramidase activity	MF
**F1SMN1**	GO:0005509	calcium ion binding	MF
**F2Z557**	GO:0000166	nucleotide binding	MF
**F2Z557**	GO:0003723	RNA binding	MF
**C6K7I0**	GO:0008565	protein transporter activity	MF
**P20112**	GO:0005509	calcium ion binding	MF
**P27594**	GO:0005525	GTP binding	MF
**P27594**	GO:0003924	GTPase activity	MF
**F1S4Y8**	GO:0003743	translation initiation factor activity	MF
**F1RSC3**	GO:0004185	serine-type carboxypeptidase activity	MF
**F1SAP4**	GO:0005524	ATP binding	MF
**F1SAP4**	GO:0004830	tryptophan-tRNA ligase activity	MF
**F1RST0**	GO:0005524	ATP binding	MF
**F2Z5K2**	GO:0004298	threonine-type endopeptidase activity	MF
**F1SLA0**	GO:0005524	ATP binding	MF
**F1SLA0**	GO:0046933	proton-transporting ATP synthase activity, rotational mechanism	MF
**F1SLA0**	GO:0046961	proton-transporting ATPase activity, rotational mechanism	MF
**F1SQ11**	GO:0005545	1-phosphatidylinositol binding	MF
**F1SQ11**	GO:0046872	metal ion binding	MF
**F1S764**	GO:0016746	transferase activity, transferring acyl groups	MF
**A5A768**	GO:0008565	protein transporter activity	MF
**F1SGP8**	GO:0005509	calcium ion binding	MF
**F1S827**	GO:0003730	mRNA 3'-UTR binding	MF
**F1SMV6**	GO:0003676	nucleic acid binding	MF
**F1SMV6**	GO:0000166	nucleotide binding	MF
**A8CYB8**	GO:0005524	ATP binding	MF
**A8CYB8**	GO:0008026	ATP-dependent helicase activity	MF
**A8CYB8**	GO:0003676	nucleic acid binding	MF
**F1SIJ9**	GO:0004648	O-phospho-L-serine:2-oxoglutarate aminotransferase activity	MF
**F1SIJ9**	GO:0030170	pyridoxal phosphate binding	MF
**F1SV06**	GO:0000166	nucleotide binding	MF
**F1SV06**	GO:0008143	poly(A) binding	MF
**F1SV06**	GO:0008266	poly(U) RNA binding	MF
**F1S9A4**	GO:0005509	calcium ion binding	MF
**F2Z5C1**	GO:0005509	calcium ion binding	MF
**F2Z5C1**	GO:0005544	calcium-dependent phospholipid binding	MF
**F1SC51**	GO:0008270	zinc ion binding	MF
**F1RI39**	GO:0005509	calcium ion binding	MF
**F1RI39**	GO:0001882	nucleoside binding	MF

Uniprot_AC	GO_Identifier	GO_Name	Aspect
**F1SJJ5**	GO:0003735	structural constituent of ribosome	MF
**F1SS24**	GO:0016504	peptidase activator activity	MF
**F1RRU7**	GO:0030246	carbohydrate binding	MF
**P33198**	GO:0004450	isocitrate dehydrogenase (NADP+) activity	MF
**P33198**	GO:0000287	magnesium ion binding	MF
**P33198**	GO:0051287	NAD binding	MF
**F1RWJ5**	GO:0008565	protein transporter activity	MF
**F1S3M9**	GO:0005198	structural molecule activity	MF
**F1SSA6**	GO:0005524	ATP binding	MF
**F1SSA6**	GO:0003774	motor activity	MF
**P28491**	GO:0005509	calcium ion binding	MF
**P28491**	GO:0030246	carbohydrate binding	MF
**P28491**	GO:0003729	mRNA binding	MF
**Q4GWZ2**	GO:0005055	laminin receptor activity	MF
**Q4GWZ2**	GO:0003735	structural constituent of ribosome	MF
**Q4GWZ2**	GO:0001618	virus receptor activity	MF
**B2CNZ7**	GO:0004197	cysteine-type endopeptidase activity	MF
**F1SIX3**	GO:0016779	nucleotidyltransferase activity	MF
**G9F6X8**	GO:0016853	isomerase activity	MF
**G9F6X8**	GO:0015035	protein disulfide oxidoreductase activity	MF
**F1RWT2**	GO:0005509	calcium ion binding	MF
**F1RGP1**	GO:0003677	DNA binding	MF
**F1RGP1**	GO:0003887	DNA-directed DNA polymerase activity	MF
**Q5MJE5**	GO:0004190	aspartic-type endopeptidase activity	MF
**Q06AT0**	GO:0005509	calcium ion binding	MF
**F1RI15**	GO:0005524	ATP binding	MF
**F1SFZ8**	GO:0005200	structural constituent of cytoskeleton	MF
**Q8SPT0**	GO:0004359	glutaminase activity	MF
**D0G0C6**	GO:0004066	asparagine synthase (glutamine-hydrolyzing) activity	MF
**D0G0C6**	GO:0005524	ATP binding	MF
**F1RS36**	GO:0005524	ATP binding	MF
**F1RS36**	GO:0043022	ribosome binding	MF
**F1S0V3**	GO:0005509	calcium ion binding	MF
**F1S0V3**	GO:0005544	calcium-dependent phospholipid binding	MF
**F1SK12**	GO:0016787	hydrolase activity	MF
**Q59IP2**	GO:0005201	extracellular matrix structural constituent	MF
**Q29092**	GO:0005524	ATP binding	MF
**F1S596**	GO:0005509	calcium ion binding	MF
**F1S596**	GO:0003723	RNA binding	MF
**P80021**	GO:0005524	ATP binding	MF
**P80021**	GO:0046933	proton-transporting ATP synthase activity, rotational mechanism	MF
**P80021**	GO:0046961	proton-transporting ATPase activity, rotational mechanism	MF
**F1RR78**	GO:0005509	calcium ion binding	MF
**B0LXK8**	GO:0004422	hypoxanthine phosphoribosyltransferase activity	MF

Table S2 KEGG pathways of differentially expressed proteins

Unipro_AC	KEGG_ID	Pathway_ID	Pathway_Name
P80021	ssc:100157880	00190	Oxidative phosphorylation
F1SLA0	ssc:100157156	00190	Oxidative phosphorylation
B0LXK8	ssc:397351	00230	Purine metabolism
D0G0C6	ssc:100312961	00250	Alanine, aspartate and glutamate metabolism
A1X898	ssc:100037299	00330	Arginine and proline metabolism
Q9TSX9	ssc:399538	00360	Phenylalanine metabolism
B0LXK8	ssc:397351	00983	Drug metabolism - other enzymes
Q4GWZ2	ssc:641351	03010	Ribosome
Q2YGT9	ssc:733592	03010	Ribosome
F2Z557	ssc:100153958	03013	RNA transport
F2Z557	ssc:100153958	03015	mRNA surveillance pathway
F1S596	ssc:100516540	04141	Protein processing in endoplasmic reticulum
P28491	ssc:100381266	04141	Protein processing in endoplasmic reticulum
Q29092	ssc:397191	04141	Protein processing in endoplasmic reticulum
F2Z5K2	ssc:497063	03050	Proteasome
F2Z557	ssc:100153958	03018	RNA degradation
F1SMN5	ssc:100518997	04010	MAPK signaling pathway
Q59IP2	ssc:397532	04151	PI3K-Akt signaling pathway
Q29092	ssc:397191	04151	PI3K-Akt signaling pathway
F1SS24	ssc:397620	04151	PI3K-Akt signaling pathway
F1SS24	ssc:397620	04512	ECM-receptor interaction
Q59IP2	ssc:397532	04512	ECM-receptor interaction
P28491	ssc:100381266	04145	Phagosome
B3XXC3	ssc:733606	04810	Regulation of actin cytoskeleton
F1SS24	ssc:397620	04810	Regulation of actin cytoskeleton
F1SMN5	ssc:100518997	04510	Focal adhesion
F1SS24	ssc:397620	04510	Focal adhesion
Q59IP2	ssc:397532	04510	Focal adhesion
Q29092	ssc:397191	04621	NOD-like receptor signaling pathway
B2LUG8	ssc:100145895	04622	RIG-I-like receptor signaling pathway
F1RI15	ssc:100524853	04612	Antigen processing and presentation
P28491	ssc:100381266	04612	Antigen processing and presentation
Q29092	ssc:397191	04915	Estrogen signaling pathway
Q29092	ssc:397191	04918	Thyroid hormone synthesis
D0G7F7	ssc:397608	04260	Cardiac muscle contraction
D0G7F7	ssc:397608	04261	Adrenergic signaling in cardiomyocytes
Q59IP2	ssc:397532	04974	Protein digestion and absorption

Table S3 Primers information

Genes	Primer senquences（5′-3′）	Products Size(bp)
GAPDH	F：GTGAAGGTCGGAGTGAACG<br>R：CTCGCTCCTGGAAGATGGTG	232
vim1	F: GAACCTGAGGGAAACCAATC<br>R: TTCGCTGCACAGAGTACATG	165
vim2	F: GTCCTCTGCCACTCTTGCTC<br>R: GCAGAAAGGCACTTGAAAGC	1640

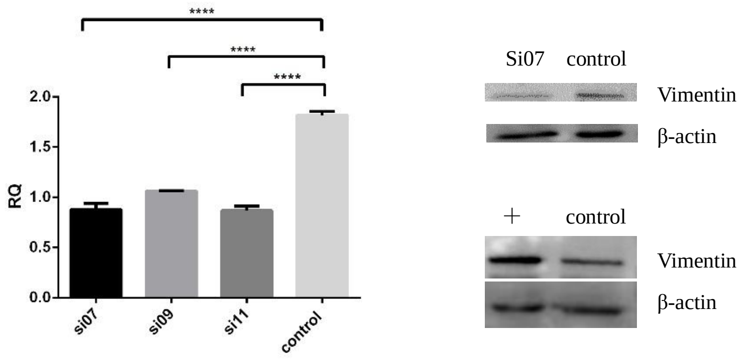

Figure S1 Results of Real-Time PCR and western-blotting of MSCs.

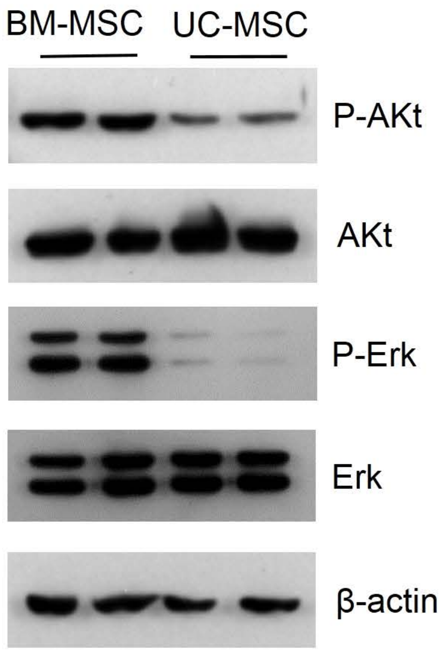

Figure S2 Phosphorylation level detection of AKt and Erk in MSCs.

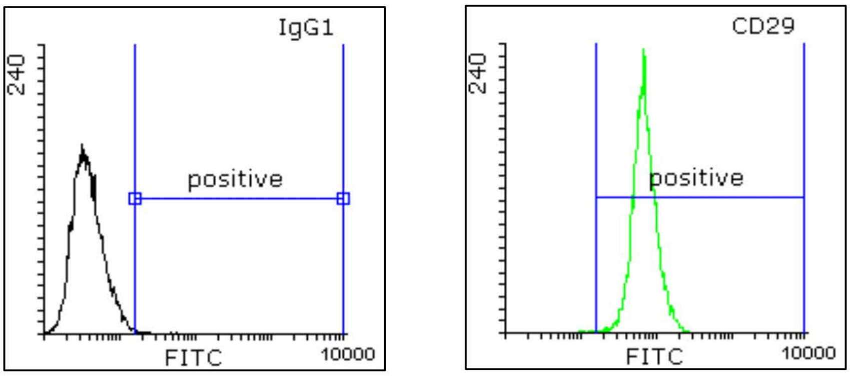

	All events: Events	positive: Events	positive: % of Vis	positive: Mean
CD29	8,922	8,895	99.70	74.63

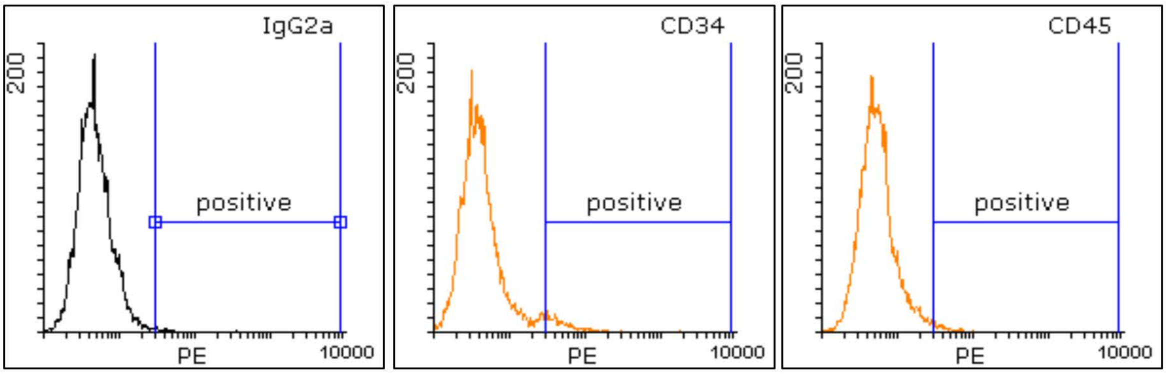

	All events: Events	positive: Events	positive: % of Vis	positive: Mean
CD34	10,000	429	4.29	98.31
CD45	10,000	128	1.28	46.31

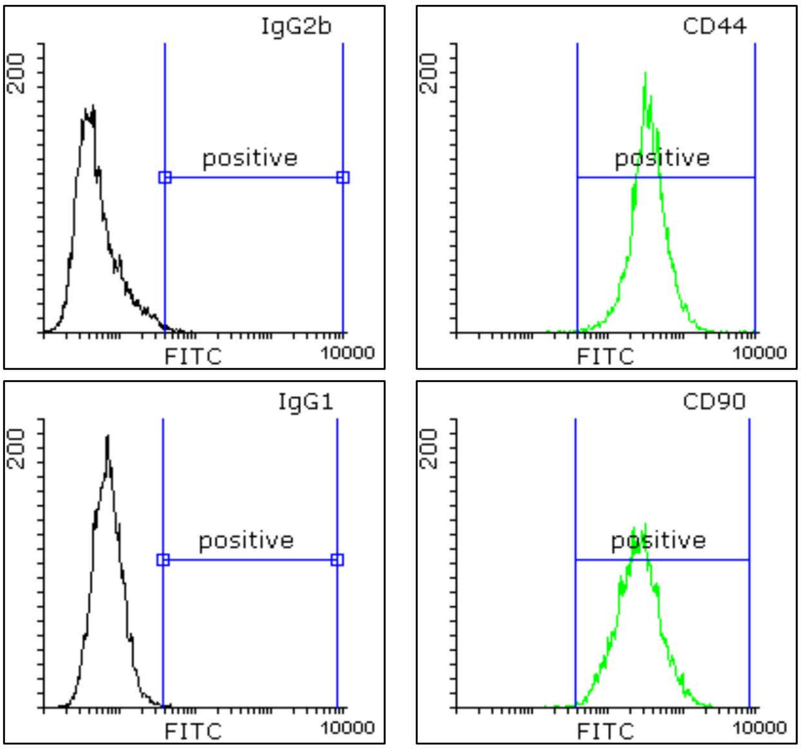

	All events: Events	positive: Events	positive: % of Vis	positive: Mean
CD44	10,000	9,965	99.65	409.59
CD90	10,000	9,959	99.59	322.02

Figure S3 Histograms of immunophenotypic profiling of BM-MSCs.

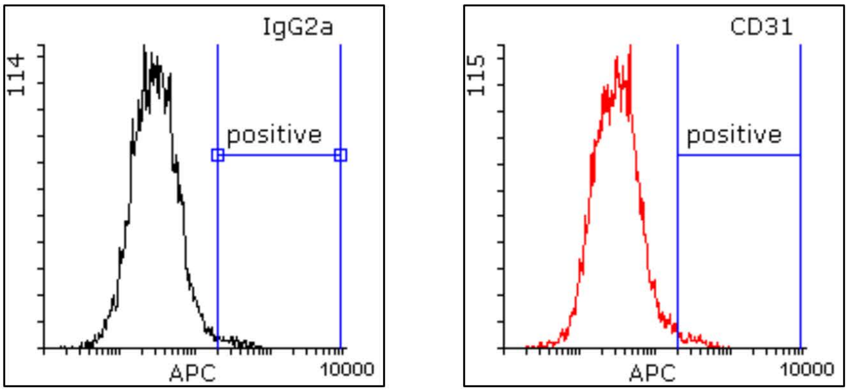

	All events: Events	positive: Events	positive: % of Vis	positive: Mean
CD31	9,963	194	1.95	403.64

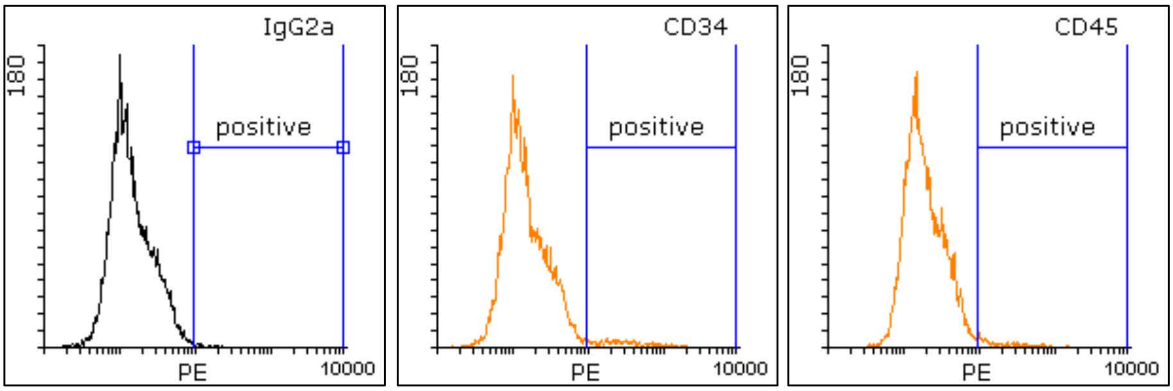

	All events: Events	positive: Events	positive: % of Vis	positive: Mean
CD34	10,000	347	3.47	453.08
CD45	10,000	228	2.28	317.32

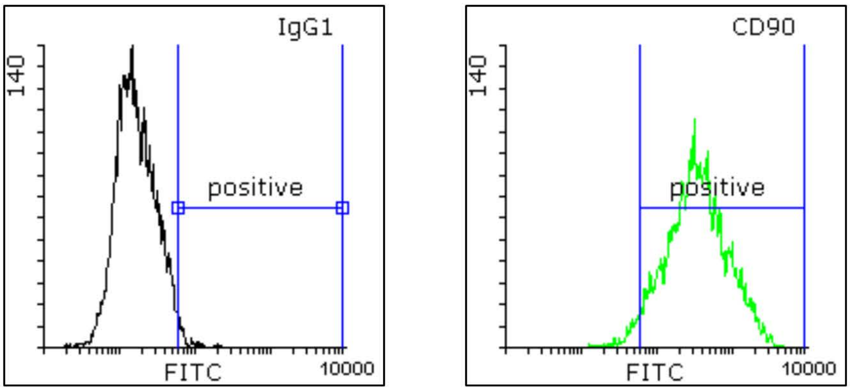

	All events: Events	positive: Events	positive: % of Vis	positive: Mean
CD90	10,000	9,647	96.47	525.42

Figure S4 Histograms of immunophenotypic profiling of UC-MSCs.
